# A multi-residue method by supercritical fluid chromatography coupled with tandem mass spectrometry method for the analysis of chiral and non-chiral chemicals of emerging concern in environmental samples

**DOI:** 10.1007/s00216-020-02780-9

**Published:** 2020-07-09

**Authors:** Jack Rice, Anneke Lubben, Barbara Kasprzyk-Hordern

**Affiliations:** 1 Department of Chemistry, Faculty of Science, University of Bath, Bath, BA2 7AY UK; 2 Material and Chemical Characterisation Facility, University of Bath, Bath, BA2 7AY UK

**Keywords:** Supercritical fluid chromatography, Environment, Mass spectrometry, Chiral chromatography, Chemicals of emerging concern

## Abstract

This manuscript presents the development, validation and application of a multi-residue supercritical fluid chromatography coupled with tandem mass spectrometry method for the analysis of 140 chiral and non-chiral chemicals of emerging concern in environmental samples, with 81 compounds being fully quantitative, 14 semi-quantitative and 45 qualitative, validated according to European Medicine Agency (EMA) guidelines (European Medicines Agency 2019). One unified LC-MS method was used to analyse all analytes, which were split into three injection methods to ensure sufficient peak resolution. The unified method provided an average of 113% accuracy and 4.5% precision across the analyte range. Limits of detection were in the range of 35 pg L^−1^–0.7 μg L^−1^, in both river water and wastewater, with an average LOD of 33 ng L^−1^. The method was combined with solid-phase extraction and applied in environmental samples, showing very good accuracy and precision, as well as excellent chromatographic resolution of a range of chiral enantiomers including beta-blockers, benzodiazepines and antidepressants. The method resulted in quantification of 75% of analytes in at least two matrices, and 56% in the trio of environmental matrices of river water, effluent wastewater and influent wastewater, enabling its use in monitoring compounds of environmental concern, from their sources of origin through to their discharge into the environment.

**Electronic supplementary material:**

The online version of this article (10.1007/s00216-020-02780-9) contains supplementary material, which is available to authorized users.

## Introduction

In 2000 the European Union (EU) set out its first Water Framework Directive (WFD) [1], which aimed to maintain and restore water quality across the EU by adopting a unified approach to discharge and emissions into surface waters. Thirty-three priority substances were identified and regulations were set up to reduce their discharge into the environment. These initial priority substances were mainly metals, flame retardants and biocides. Additional compounds were prioritised by a new directive in 2012 [2]. As part of this expansion, the NSAID diclofenac and the synthetic oestrogens 7β-oestradiol (E2) and 17α-ethinyloestradiol (EE2) were proposed as potential priority substances. In 2019 the publication of the EU’s ‘strategic approach to pharmaceuticals in the environment’ [3] mapped out the EU’s objectives for how to monitor and reduce the usage of pharmaceuticals, promote ‘greener’ manufacturing, improve environmental risk assessments (ERAs) for pharmaceuticals and support monitoring of these compounds in fresh and coastal waters. However, despite this focus on pharmaceuticals as chemicals of emerging concern (CECs) in the environment, there has been no assessment on the effect of chirality. The effects of chirality in pharmaceuticals for human consumption are well documented, particularly in the wake of the thalidomide disaster; however, the effect of chirality on environmental toxicity is still not as well understood. Both new and existing ERAs currently do not require any examination of the effect of chirality on their environmental toxicity [4, 5]. The main reason for not acknowledging chirality in ERAs is a lack of published knowledge on the relative effects of enantiomers, as well as lack of methods enabling research in this area. New, sensitive, multi-residue methods enabling identification and quantification of chiral and non-chiral CECs within one analytical method are therefore urgently needed.

Methods have already been developed for the monitoring of CECs in surface waters [6–18] and their effect on the aquatic environment [19–21]. However, many of these methods are not performed using chiral discrimination as separation at enantiomeric level poses an analytical challenge. Enantiomers cannot be separated by conventional reversed-phase chromatography utilising C18 stationary phases. Instead specific chiral chromatography methods must be developed utilising expensive chromatography columns. The types of chiral selectors used range from small molecules like modified benzenes to macrocycles and proteins. The difficulty of chiral chromatography is that the biological nature of many chiral selectors restricts the range of solvents and other modifiers which can be used in the methods [6, 22]. This generally also restricts the ability to develop LC methods with mobile phase gradients, thus increasing analytical runtimes. Chiral columns usually operate within inefficient HPLC modes due to large silica particle sizes being used.

Supercritical fluid chromatography (SFC) is an increasingly popular analytical technique that is of great interest for chiral analysis [23, 24]. As with other chiral chromatography, chiral-SFC requires a stationary phase containing a chiral selector, but unlike reversed-phase (RP) chiral-LC, this chiral selector is a small chiral molecule, rather than a biomolecule, enabling the use of a wider range of solvents. Additionally, these columns can tolerate higher backpressures and have shorter equilibration times making the use of mobile phase gradients possible using SFC. SFC generally uses CO_2_ as its primary mobile phase [25] with an organic co-solvent for elution such as n-heptane, isopropyl alcohol or methanol. This makes SFC a ‘greener’ analytical technique compared to traditional chromatography because of the use of renewable CO_2_ as a mobile phase. Acidic or basic modifiers, such as formic acid or ammonium hydroxide, are also commonly added to help limit unwanted analyte-stationary phase interactions, such as those with uncapped silanols on unmodified silica stationary phases. The main requirement for using SFC is that analytes have to be soluble in either the supercritical CO_2_ or the organic co-solvent. Fortunately, most CECs are soluble in common organic solvents like methanol or acetonitrile [14, 24, 26].

(Stereoselective) analysis of trace levels of CECs is also required in wastewater-based epidemiology (WBE). Large public health monitoring studies have become increasingly widespread, in terms of both the size of the populations studied [27–31] and the range of analytes that have been detected which are often those of interest as environmental micropollutants [27, 32–40]. However, despite many of these analytes having at least one chiral centre, most published WBE analytical methods do not include enantiomer separations. Whilst desirable, this trend can be limiting as it effectively excludes analytical approaches that require long sample preparation or analysis times, like chiral HPLC. Therefore, perhaps the greatest benefit of SFC is that it could be used for the analysis of both public and environmental health determinants. Hence, this manuscript aims to deliver a rapid method with sensitive and selective multi-residue measurements of structurally variable groups of CECs.

## Materials and methods

### Materials

High-purity (≥ 99.97%), food-grade, gaseous CO_2_ was supplied to the system from an unheated cylinder without a dip tube. All solvents, except water, were of MS grade and purchased from VWR. Ultrapure water was obtained from a MilliQ purification system (Merck Millipore, UK). Mobile modifiers triflouroacetic acid (TFA), formic acid (FA), ammonium hydroxide, ammonium formate and ammonium acetate were all purchased from Sigma-Aldrich UK. A full list of analytes and internal standards, and their associated supplier information, is provided in the Electronic Supplementary Material (ESM, Table S1). All glassware used was silanised with dimethylchlorosilane (DMDCS in toluene, Sigma-Aldrich) prior to use to limit adsorption of basic analytes to silanol sites on the surface of the glassware. Solid-phase-extraction cartridges used for validation were Oasis, 60 mg, 3 cc, HLB SPE cartridges (Waters, UK). Sample evaporation was performed using a turbovap LV concentration workstation (Caliper, UK). Whatman GF/F filter papers were used for all water sample filtrations.

### Methods

A Waters Acquity UltraPerformance Covergence Chromatography (UPC^2^) instrument coupled with either a Waters Acquity QDa single quadrupole mass detector or a Waters Xevo TQD triple quadrupole mass spectrometer was used for method development. Three Waters chromatography columns were tested: (i) Waters Trefoil 2.1 × 50-mm, 2.5-μm amylose-based column modified with 3,5-dimethylphenylcarbamate (AMY1); (ii) Waters Trefoil 2.1 × 50-mm, 2.5-μm cellulose-based column modified with 3,5-dimethylphenylcarbamate (CEL1); and (iii) Waters Trefoil 2.1 × 50-mm, 2.5-μm cellulose-based column modified with 3-chloro-4-methylphenylcarbamate (CEL2).

#### UPC^2^-MS method development

##### Initial screening

Initial screening used conditions outlined in the Waters chiral method development strategy for optimal path screening [41]. A range of analytes were selected for this initial screening including both chiral and achiral compounds, with a full listing provided in the ESM (Table S1). The following four methods were trialled:

(i) AMY1 (column) with A: 100% CO_2_ and B: 1:1:1 (v/v) EtOH:IPA:MeCN with 20 mM NH_4_OAc
(ii) CEL1 (column) with A: 100% CO_2_ and B: 1:1 (v/v) MeOH:IPA with 0.2% (v/v) TFA
(iii) CEL2 (column) with A: 100% CO_2_ and B: 1:1 (v/v) EtOH/MeCN with 0.2% (v/v) TFA
(iv) AMY1 with A: 100% CO_2_ and B: 1:1 (v/v) EtOH:IPA with 0.2% (v/v) TFA

All columns were 2.1 × 50-mm Trefoil chiral columns with a particle size of 2.5 μm. Initial conditions were 3% B mobile phase at a flow rate of 1.2 mL min^−1^, a column temperature of 40 °C ± 5 °C, an automatic backpressure regulator pressure of 3200 psi and a sample injection volume of 2 μL. All samples were dissolved in methanol. The QDa detector was set to alternating positive and negative ESI mode and collected data in both modes for a mass range of 150–650 *m*/*z*. A mobile-phase gradient was applied in all methods and consisted of holding the initial 3% B mobile phase conditions for 0.5 min, before increasing the % B mobile phase to 60% B over 1.5 min and then holding at 60% B for 0.5 min. After this, initial conditions were restored by decreasing the mobile phase B % to 3% B over 0.5 min and holding at this level for 0.5 min before starting the next run. The total cycle time for each sample was 3.5 min, with MS data collected for 3.0 min. The make-up solvent used was 9:1 (% v:v) MeOH:H_2_O with 1% formic acid and a flowrate of 0.45 mL min^−1^. Analyte detection was performed using single-quadrupole MS in ESI+ and ESI− scanning mode using the following conditions: ESI+ and ESI− scan conditions used a centroid data format and scanned between 120 and 650 *m*/*z* over 3.5 min with a cone voltage of 15 V. ESI+ capillary voltage was 1.5 kV; ESI− capillary voltage was 0.8 kV. The initial optimal screening path was used to identify column chemistries and mobile phases that could result in at least partial separation of chiral analytes, as well as the detection of both chiral and achiral analytes.

##### Selection of mobile phases

Following initial screening, four mobile-phase compositions (A–D) were selected and tested with three different columns (see ESM Tables S2 and S3 for details). Analytes were detected using the QDa single-quadrupole MS using a targeted ESI+ MS method rather than a scanning method. Using these methods, the following chiral analytes were analysed at a concentration of 100 μg L^−1^: atenolol, bisoprolol, ketamine, metoprolol, propranolol, temazepam, zolpidem, amitriptyline, MDMA, methamphetamine, amphetamine, PMA, mephedrone, venlafaxine, desmethylvenlafaxine, cocaine, benzoylecognine, methadone, EDDP, fluoxetine, chloramphenicol and tramadol.

#### Solid-phase extraction

To enable the sensitive analysis of environmental samples, a solid-phase extraction (SPE) method was developed for use with influent wastewater, effluent wastewater and river water. Prior to extraction, samples were homogenised by inverting their storage containers to resuspend settled sediment. Fifty millilitres of each sample solution was then taken and 25 μL of a 2-mg L^−1^ internal standard stock solution was added. The sample was then filtered through Whatman GF/F filter paper and stored on ice before extraction. Waters Oasis HLB cartridges were used to extract samples and were conditioned according to the manufacture’s guidelines, using 2 mL of methanol followed by 2 mL of ultrapure water. The samples were loaded onto the SPE cartridges under vacuum at a rate of 3 mL min^−1^ before washing with 3 mL of ultrapure water. The samples were then left to dry under vacuum for 30 min. Once dried, the samples were eluted or directly stored at − 20 °C for future analysis. The samples were eluted using 4 mL of MeOH into silanised glass tubes before being placed in a water bath at 30 °C and evaporated in a turbovap evaporator under a gentle stream of N_2_. Once completely evaporated, the samples were reconstituted to a final volume of 500 μL in 100% MeOH.

#### Method performance

Validation was carried out in accordance with recommendations set by the European Medicines Agency (EMA) [42, 43]. Method validation was performed in 100% methanol, in influent wastewater and in river water. The following parameters were evaluated: instrument and method accuracy, precision, linearity and range, limits of detection and quantification recovery, and signal suppression.

A mixture of all available analytes was prepared from stock solutions at a concentration of 2 mg L^−1^ of each analyte in methanol, and was used to create all working solutions, spiked samples and quality controls as described below. Another mixture of deuterated compounds was also prepared at the same concentration (2 mg L^−1^ of each in methanol) and is described in the text as internal standards or internal standard mixture. The internal standard mixture was used to create all working solutions, spiked samples and quality controls as described below.

##### UPC^2^-TQD instrument method performance

The instrument linearity and concentration range over which an analyte could be detected were determined using a seventeen-point calibration curve ranging from 0.0 to 1000 μg L^−1^ for achiral analytes and 0.0 to 500 μg L^−1^ for individual chiral isomers such that the concentration of the sum of both isomers covered a range of 0.0–1000 μg/L. Calibrant concentrations used are as follows, with chiral concentrations in brackets: 0 (0) μg L^−1^, 0.01 (0.005) μg L^−1^, 0.05 (0.025) μg L^−1^, 0.1 (0.05) μg L^−1^, 0.5 (0.25) μg L^−1^, 1 (0.5) μg L^−1^, 5 (2.5) μg L^−1^, 10 (5) μg L^−1^, 25 (12.5) μg L^−1^, 50 (25) μg L^−1^, 100 (50) μg L^−1^, 200 (100) μg L^−1^, 400 (200) μg L^−1^, 600 (300) μg L^−1^, 800 (400) μg L^−1^ and 1000 (500) μg L^−1^. One hundred micrograms per litre of internal standard was included in each calibrant sample. Instrument linearity was determined using the *R*^2^ of a linear line of best fit, as determined by the data analysis software used (MassLynx V4.1). The linear range was the calibrant concentration range over which the linearity was calculated. Two microlitres of each calibrant sample was injected three times for the determination of linearity.

Instrument limits of detection (iLOD) and quantification (iLOQ) were determined using the calibration curve. iLOD was determined as the lowest measured calibrant concentration with an average peak signal to noise ratio (*S*/*N*) of greater than or equal to 3 (*S*/*N* ≥ 3) across three repeat calibrant injections. iLOQ was determined as the lowest measured calibrant concentration with an average *S*/*N* ≥ 10 across three repeat calibrant injections.

Accuracy was determined at three different concentrations for both chiral and achiral analytes. For achiral analytes, accuracy was determined at 10, 50 and 200 μg L^−1^, whilst for chiral analytes, accuracy was determined at 5, 25 and 100 μg L^−1^. Samples were injected in triplicate and accuracy at each concentration (*x*) was calculated using the following equation (Eq. 1), where *x* is the theoretical concentration, e.g. 10 μg L^−1^, and *x*_1–3_ is the concentration measured in each sample.

1 $$ \mathrm{accuracy}\ \left(\%\right)=\frac{x}{\mathrm{average}\ \left({x}_1,{x}_2,{x}_3\right)}\ast 100\kern8em $$

Precision was determined at the same concentrations as those used for the accuracy and determined as the relative standard deviation (RSD) between triplicate injections at each concentration (Eq. 2). Interim or interday precision was calculated by determining precision using the triplicate injection of freshly prepared samples on two different, non-sequential days. Repeatability was measured as the average RSD of each day’s precision.

2 $$ \mathrm{intraday}\ \mathrm{precision}\ \left(\%\right)=\frac{\sigma_{x_1-{x}_3}}{\mathrm{average}\left({x}_1,{x}_2,{x}_3\right)}\ast 100\kern4.25em $$

Relative retention time (*t*_rel_) was measured as the difference between the analyte’s peak retention time (*t*_A_) in mobile phase and the peak retention time of its assigned internal standard (*t*_ISTD_) (Eq. 3).

3 $$ {t}_{\mathrm{rel}}=\kern0.5em \frac{t_A}{t_{\mathrm{ISTD}}}\kern4.75em $$

Enantiomeric resolution (*R*_*s*_) was also calculated (Eq. 4). The base peak width (*w*_*x*_*S*^−1^) for each enantiomer was calculated as the difference between the average peak-end and peak-start times. *t*_n_ refers to the peak top retention time of the peak, with the subscript referring to the order in which the peaks eluted in.

4 $$ {R}_s=\kern0.5em \frac{t_2-{t}_1}{0.5\left({w}_2+{w}_1\right)}\kern1.25em $$

Enantiomeric fraction (EF) was measured as the relative concentration of the first eluting enantiomer (*E*_1_) relative to the sum of the concentration of both enantiomers (Eq. 5)

5 $$ \mathrm{EF}=\frac{\left[{E}_1\right]}{\left[{E}_1\right]+\left[{E}_2\right]}\kern2em $$

##### SPE-UPC^2^-TQD validation

Signal suppression (SS) was calculated by comparing analyte peak areas in river water (RW) or wastewater (WW) matrix to equivalent peak areas in the mobile phase (Eq. 6). Samples of each matrix underwent filtration and SPE as described above, but without addition of internal standards. After matrix elution, 50 ng of each internal standard and 50 ng of each analyte were added.

6 $$ \mathrm{signal}\ \mathrm{suppression}=1-\left(\frac{\mathrm{analyte}\ \mathrm{peak}\ \mathrm{area}\ {\frac{\mathrm{RW}}{\mathrm{WW}}}_{\mathrm{SS}}-\mathrm{analyte}\ \mathrm{peak}\ \mathrm{area}\ {\frac{\mathrm{RW}}{\mathrm{WW}}}_{\mathrm{blank}}}{\mathrm{analyte}\ \mathrm{peak}\ \mathrm{area}\ {\mathrm{QC}}_{100}}\right)\ast 100\kern2.25em $$

Absolute and relative recoveries were calculated by comparison of analyte peak areas or concentrations in river water (RW) or influent wastewater (WW), to analyte peak areas or concentrations in the mobile phase (Eq. 7). Both recoveries were determined in triplicate at three different concentrations, and then averaged. Analyte was spiked into samples of matrix to give concentration of 0, 0.11, 0.5 or 2 μg L^−1^, along with 50 ng of each internal standard, before filtration and SPE as described above. Analyte concentrations were calculated using calibration curves prepared as outlined above, and internal standards assigned by using a combination of similar retention times (*t*_rel_ ≈ 1) and signal suppression (SS_analyte_ ≈ SS_ISTD_) factors.

7 $$ \mathrm{absolute}\ \mathrm{recovery}\ \left(\%\right)=\frac{\mathrm{analyte}\ \mathrm{peak}\ \mathrm{area}\ {\frac{\mathrm{RW}}{\mathrm{WW}}}_x-\mathrm{analyte}\ \mathrm{peak}\ \mathrm{area}\ {\frac{\mathrm{RW}}{\mathrm{WW}}}_0}{\mathrm{analyte}\ \mathrm{peak}\ \mathrm{area}\ {\mathrm{QC}}_x}\ast 100 $$

Method accuracy and precision were calculated at 0.05, 0.5 and 2 μg L^−1^ by spiking each analyte into either RW or WW at these concentrations. Method accuracy was calculated (Eq. 8) to determine how close the measured concentration (analyte conc. RW/WW_*x*1–*x*3_) was to spiked concentrations (*x*), whilst method precision (Eq. 9) was used to measure how similar the measured concentration values were to each other. For each equation, the concentration of analyte in the blank RW or WW samples (analyte conc. RW/WW_0_)_*x*1–*x*3_ was subtracted from the measured concentration, to account for analyte already present in the matrix. The standard deviation of RW/WW concentration is denoted by *σ*.

8 $$ \mathrm{method}\ \mathrm{accuracy}\ \left(\%\right)=\frac{x}{\mathrm{average}\ {\left(\mathrm{analyte}\ \mathrm{conc}.{\frac{\mathrm{RW}}{\mathrm{WW}}}_x-\mathrm{analyte}\ \mathrm{conc}\ {\frac{\mathrm{RW}}{\mathrm{WW}}}_0\right)}_{x1-x3}}\ast 100 $$

9 $$ \mathrm{method}\ \mathrm{precision}\ \left(\%\mathrm{RSD}\right)=\frac{\sigma {\left(\mathrm{analyte}\ \mathrm{conc}.{\frac{\mathrm{RW}}{\mathrm{WW}}}_x-\mathrm{analyte}\ \mathrm{conc}\ {\frac{\mathrm{RW}}{\mathrm{WW}}}_0\right)}_{x1-x3}}{\mathrm{average}\ {\left(\mathrm{analyte}\ \mathrm{conc}.{\frac{\mathrm{RW}}{\mathrm{WW}}}_{\mathrm{x}}-\mathrm{analyte}\ \mathrm{conc}\ {\frac{\mathrm{RW}}{\mathrm{WW}}}_0\right)}_{x1-x3}}\ast 100 $$

Enantiomeric fraction and chiral peak resolution were calculated at 0.05, 0.50 and 2 μg L^−1^ using Eq. 4 and Eq. 5 respectively, in both river water and wastewater.

Method LOD (mLOD) and method LOQ (mLOQ) were calculated using the instrument LOD (iLOD) and instrument LOQ (iLOQ) and the average relative recovery (Rec_average_) (Eq. 10). mLOQ was also calculated using Eq. 10 by substituting iLOD for iLOQ. CF is the concentration factor of the SPE method described above, which is calculated as the initial volume of matrix used (50 mL) divided by the final sample volume (0.5 mL).

10 $$ \mathrm{mLOD}=\frac{\mathrm{iLOD}\ast 100}{{\mathrm{Rec}}_{\mathrm{Average}}\ast \mathrm{CF}}\kern1.25em $$

#### Analysis of environmental samples

Samples of influent, effluent and river water were collected by grab sampling to test the suitability of the method in the analysis of real samples. Influent and effluent grab samples were collected on the same day, at the same time, from a wastewater treatment plant (WWTP) serving a city in the South-West of the UK which discharges its effluent into a river. River water grab samples were collected mid-stream, upstream and downstream of the WWTP where influent and effluent samples were also collected. All samples were transported back to the lab on ice in separate HDPE containers, and prepared as described in 2.2.1.4. Two samples were prepared for each matrix, and each sample was injected and analysed in triplicate. Enantiomeric fractions of chiral analytes were calculated using Eq. 5. Two rounds of samples were collected. The first set of environmental samples were collected in early January 2018 and were used for method validation, as detailed above, whilst the second set were collected in February 2018 and analysed for as proof of concept (3.3).

## Results and discussion

### Method development

#### Initial screening

Results from the initial screening using conditions described in ‘UPC2-MS method development’ can be generalised as follows: (1) Amphetamine and MDMA’s enantiomers were not separated by any of the four methods. Ketamine was partially or fully separated by all but the CEL-1 method. (2) CEL-1 was the only method to partially separate methamphetamine and mephedrone. (3) CEL-2 was able to only partially separate venlafaxine and ketamine. (4) No combination of methods was able to separate all chiral analytes.

#### Selection of mobile phase composition

Table S4 (see ESM) shows the enantioselective separation that was achieved when using the conditions described in Tables S2 and S3 (see ESM) to separate a range of chiral pharmaceuticals and drugs of abuse. The most consistent and best-performing method was method B2, using CEL-1 and the following mobile phase composition: mobile phase A: CO_2_ and mobile phase B: 1:1:1 (v/v) MeOH:MeCN:IPA at a flow rate of 1.5 mL min^−1^ with a total runtime of 9 min. Mass chromatograms showing the enantioselective separation are presented in Fig. S1 (see ESM). This method was considered as performing best and was therefore selected for validation. The analytical set-up was altered to allow for coupling the SFC to the triple-quadrupole instrument. This was achieved by installing a splitter on the SFC instrument, post-dilution with make-up solvent, channelling the flow into the switching valve of the adjoining Xevo TQD instrument instead of into the QDa module of the UPC^2^. This transition to the new instrument also necessitated adaptations to the mobile-phase conditions due to higher system backpressures. The mobile-phase flow rate was therefore decreased to 0.75 mL min^−1^ with all other chromatography conditions left unaltered. The MS conditions used were as follows: capillary voltage 3.0 kV, desolvation temperature 400.0 °C, source temperature 150.0 °C and cone gas flow 100.0 L min^−1^. The MRM transitions, cone voltages and collision energies used with the TQD instrument are detailed in ESM Table S5. In total 210 compounds were analysed using method B2, necessitating their division into multiple MS methods. First the analytes and deuterated internal standards ionised under negative ESI conditions were selected for inclusion in the NEG method. The remaining analytes were then separated into two groups to create a total of three methods, all with a similar number of scan points per analyte peak. The methods can be summarised as follows: (i) DAC method: drugs of abuse, antibiotics and chiral analytes, and associated deuterated internal standards ionised in ESI+ mode; (ii) PHARMA method: pharmaceuticals, pesticides and other analytes, and associated internal standards ionised in ESI+; and (iii) NEG method: analytes ionised in ESI− (ESM Table S6).

### Method validation

#### UPC^2^-TQD validation

Instrument linearity, limits of detection and quantification are shown in ESM Table S7. Internal standards were assigned using analyte and internal standard retention time, signal suppression and absolute recovery factors, which are shown in the ESM (Table S8), with priority given to (in order) relative retention time and analyte signal suppression in wastewater and river water. EMA guidelines were used to determine which compounds were fully quantitative, with compounds that did not meet the required specifications being described as semi-quantitative or qualitative, using criteria discussed below.

Linearity results were generally excellent, with most analytes showing a calibration *R*^2^ value > 0.997, with only fexofenadine, iopromide, O-desmethyl naproxen and triclosan being considered as semi-quantitative due to an *R*^2^ between 0.990 and 0.997. Semi-quantitative compounds appear in italics in Table S7 (see ESM). The linearity results were used to create a calibration curve to quantify each of the analytes relative to its assigned internal standard. Instrument accuracy and precision were then measured on three non-consecutive days over the course of a week, with new samples prepared each day. Results of average instrument accuracy and precision determinations at three concentrations are shown in Table S9 (see ESM).

Accuracy at each concentration should be 100% ± 20%, and where results are outside of this limit, they were recorded in italics in Table S9 (see ESM). This deviation largely occurred in the 10 μg L^−1^ samples. Compounds in italics are considered to be semi-quantitative. Precision was recorded as required < 20% RSD for most analytes. Precision of > 20% occurred mostly in analytes at 10 μg L^−1^. The instrument’s ability to resolve enantiomers was assessed along with the enantiomeric fraction in the mobile phase, which should be close to 0.5. Resolution and EF were calculated at 10, 50 and 200 μg L^−1^ and the average results are shown in Table 1. The resolutions are all greater than 1.2 and therefore sufficient for quantification [44, 45]. E2-tramadol was used to calculate resolution but not EF as it could not be successfully validated. Figures S2–S4 (see ESM) show the extracted ion chromatograms obtained for each analyte in mobile phase at a concentration of 100 μg L^−1^ using the MRM1 transitions detailed in ESM Table S5. Figures S2–S4 (see ESM) were broken down alphabetically by the MS method used, as described in ESM Table S6.

**Table 1:** Enantiomeric fraction and peak resolution for chiral analytes (*n* = 12)

Analytes	Mobile phase			
	EF	SD	*R*_*s*_	SD
10,11-Dihydro-10-hydroxycarbamazepine	0.5	0.02	10.5	0.8
Alprenolol	0.5	0.01	5.6	0.1
Atenolol	0.5	0.02	26.3	2.5
Bisoprolol	0.5	0.02	12.2	3.3
Metoprolol	0.5	0.01	12.6	2.5
Mirtazapine	0.5	0.01	8.7	0.2
Oxazepam	0.5	0.02	15.5	2.1
Propanolol	0.5	0.07	18.2	3.3
Tramadol	–	–	8.6	1.6

#### SPE-UPC^2^-TQD validation

Average relative SPE-UPC^2^-TQD method recovery was determined at three concentrations (0.1, 0.5 and 2 μg L^−1^) and presented in Table 2 as averages. Full recoveries at each concentration are presented elsewhere (ESM Tables S8 and S10). Signal suppression was calculated at 1 μg L^−1^ only.

**Table 2:** Average relative method recovery at three concentrations (*n* = 9) and signal suppression (*n* = 3) in river water and wastewater (semi-quantitative compounds are presented in italics)

Analyte	Average relative recovery				Signal suppression (%)			
	River water		Wastewater		River water		Wastewater	
	%	SD	%	SD	%	SD	%	SD
Aminorex	58	10.2	120	9.4	12	0.07	− 23	0.07
Anhydroecgonine methylester	97	5.9	108	1.1	− 17	0.05	− 14	0.05
Benzophenone-1	60	9.3	102	18.6	− 42	0.12	− 82	0.11
Benzophenone-4	97	3.6	89	2.3	− 51	0.23	− 21	0.16
Benzoylecgonine	101	10.6	88	16.9	− 3	0.02	− 2	0.02
*Benzylpiperizine*	*37*	*8.9*	*49*	*2.2*	*46*	*0.04*	*33*	*0.03*
Bezafibrate	66	12.5	69	7.1	− 17	0.05	− 17	0.04
Buprenorphine	53	10.6	61	5.2	1	0.12	30	0.12
*Candesartan cilexetil*	*47*	*19.0*	*71*	*16.6*	− *4*	*0.04*	− *82*	*0.06*
Carbamazepine	90	9.9	89	6.8	− 1	0.03	1	0.03
Carbamazepine 10,11 epoxide	86	12.7	90	18.8	− 2	0.03	− 18	0.04
*Carprofen*	*62*	*0.9*	*47*	*8.2*	− *10*	*0.00*	*2*	*0.00*
Citalopram	83	8.2	57	11.8	4	0.05	18	0.05
Clothiniadin	79	13.5	104	10.4	− 11	0.04	− 30	0.03
Cocaethylene	90	6.6	95	1.6	− 9	0.01	− 3	0.02
Cocaine	89	0.8	90	3.1	− 3	0.02	4	0.02
Codeine	118	30.0	68	15.6	− 3	0.06	16	0.08
Cotinine	109	10.9	77	11.7	− 2	0.03	10	0.05
Desmethylcitalopram	76	15.0	125	15.4	35	0.03	− 60	0.04
*DHMA*	*82*	*4.2*	*54*	*7.4*	*17*	*0.07*	*50*	*0.12*
Diazepam	84	9.1	93	2.9	− 4	0.05	− 4	0.03
Diazinon	65	11.5	122	15.1	− 10	0.06	− 82	0.12
Diclofenac	82	2.4	75	14.7	− 28	0.02	− 24	0.04
Dihydrocodeine	87	2.6	81	3.4	− 1	0.02	7	0.08
Dihydroketoprofen	86	11.7	57	18.1	− 16	0.11	− 2	0.08
Dihydromorphine	90	3.4	88	17.5	4	0.04	18	0.03
*Diltiazem*	*75*	*6.5*	*68*	*30.6*	*21*	*0.01*	*55*	*0.02*
*Duloxetine*	*24*	*3.6*	*49*	*5.1*	*48*	*0.04*	*11*	*0.05*
E1-10,11-Dihydro-10-hydroxycarbamazepine	111	14.3	112	13.7	− 12	0.01	1	0.04
E1-Alprenolol	83	13.8	66	5.0	2	0.05	33	0.05
E1-Atenolol	93	7.3	95	1.4	− 5	0.03	4	0.04
E1-Bisoprolol	87	10.1	89	18.1	− 7	0.03	− 7	0.02
E1-Metoprolol	87	11.3	80	14.7	− 6	0.05	1	0.05
E1-Mirtazapine	80	8.1	78	2.8	2	0.05	15	0.05
E1-Oxazepam	85	7.0	79	12.8	2	0.06	8	0.04
E1-Propanolol	89	6.9	85	10.0	− 2	0.03	− 6	0.04
*E1-Tramadol*	*89*	*19.9*	*37*	*11.1*	*3*	*0.06*	*55*	*0.04*
E2-10,11-Dihydro-10-hydroxycarbamazepine	101	5.6	113	7.2	− 8	0.03	− 4	0.02
E2-Alprenolol	69	11.8	72	1.9	5	0.09	19	0.07
E2-Atenolol	88	9.8	95	1.1	− 5	0.03	4	0.03
E2-Bisoprolol	86	9.5	90	3.5	− 1	0.04	− 1	0.04
E2-Metoprolol	82	14.6	93	6.5	5	0.03	2	0.03
E2-Mirtazapine	80	10.3	80	0.2	1	0.02	9	0.02
E2-Oxazepam	94	5.0	79	9.4	4	0.06	6	0.05
E2-Propanolol	93	2.4	87	1.1	2	0.06	2	0.06
Ethylparaben	91	11.6	87	4.5	− 38	0.08	− 59	0.07
*Fexofenadine*	*89*	*8.4*	*50*	*15.1*	− *1*	*0.06*	*21*	*0.08*
Griseofulvin	88	5.0	84	11.0	4	0.05	22	0.04
Heroin	88	3.9	87	2.3	− 7	0.04	− 7	0.06
HMA	59	5.4	77	3.8	15	0.02	− 6	0.02
*HMMA*	*81*	*8.8*	*147*	*12.1*	− *3*	*0.04*	− *52*	*0.04*
Hydrocodone	94	6.6	74	8.1	8	0.01	11	0.04
Imatinib	68	5.8	100	19.1	− 9	0.02	− 42	0.02
Imidacloprid	104	2.9	137	17.1	− 24	0.03	− 63	0.02
Indoprofen	79	10.9	70	4.9	4	0.01	24	0.03
*Iopromide*	*88*	*10.1*	*163*	*13.3*	*15*	*0.05*	− *139*	*0.05*
Ketamine	92	17.3	104	7.2	− 15	0.04	− 11	0.04
Ketoprofen	94	29.2	68	19.2	− 86	0.17	− 85	0.16
MDA	70	9.5	89	14.0	− 9	0.02	3	0.03
MDMA	81	10.2	82	11.2	− 4	0.04	4	0.04
MDPV	75	9.5	87	4.0	− 2	0.04	− 1	0.03
*Memantine*	*95*	*19.3*	*161*	*9.3*	− *14*	*0.05*	− *19*	*0.04*
Mephedrone	76	14.9	62	8.3	14	0.04	38	0.03
Metazachlor	96	4.4	83	14.0	14	0.05	13	0.05
Methadone	79	9.8	78	4.8	− 6	0.07	4	0.05
Methamphetamine	80	0.4	90	5.2	9	0.04	13	0.04
Methylparaben	99	0.9	102	1.6	− 63	0.11	− 73	0.11
Morphine	98	3.1	102	6.9	1	0.06	11	0.08
Nordiazepam	84	7.4	96	4.0	− 9	0.03	− 6	0.03
Norephedrine	82	6.1	72	9.4	1	0.02	− 13	0.03
Normorphine	51	6.9	66	5.6	32	0.04	29	0.04
Nortriptyline	62	1.7	71	13.2	7	0.06	3	0.05
O-Desmethylnaproxen	72	11.0	88	33.9	− 23	0.00	− 50	0.00
Omeprazole	95	11.8	132	13.2	1	0.04	− 48	0.06
Oxadiazon	65	16.8	74	18.9	22	0.06	0	0.07
Oxycodone	91	4.2	104	13.7	− 3	0.03	4	0.03
*Oxymorphone*	*13*	*3.6*	*45*	*6.8*	*81*	*0.05*	*52*	*0.05*
Pholcodine	90	3.4	109	8.9	− 18	0.04	− 39	0.12
Praziquantrel	85	9.9	96	5.5	− 3	0.07	− 2	0.04
Propylparaben	73	35.5	88	22.5	− 81	0.07	− 113	0.06
Quetiapine	82	4.9	106	23.5	− 1	0.03	− 2	0.02
Risperidone	81	5.0	93	5.4	6	0.03	− 10	0.04
Salbutamol	91	9.8	94	8.5	− 5	0.02	− 48	0.04
Sotalol	78	9.2	146	3.7	10	0.02	− 83	0.04
Sulphadiazine	57	7.3	70	5.6	12	0.04	13	0.05
Sulphamethoxazole	119	14.2	119	1.8	17	0.04	− 12	0.04
Sulphapyridine	87	6.8	111	9.9	17	0.04	9	0.04
Terbutaline	99	0.2	84	1.5	99	0.01	12	0.01
Terbuthylazine	75	5.0	87	9.6	13	0.06	10	0.07
Tetramisole	96	6.8	93	0.2	3	0.05	5	0.07
*Thiamethoxam*	*113*	*6.3*	*149*	*15.4*	− *40*	*0.03*	− *58*	*0.04*
*Triclosan*	*90*	*38.6*	*36*	*5.1*	− *59*	*0.00*	− *187*	*0.00*
Valsartan	80	4.2	74	3.5	− 16	0.05	− 8	0.04
Vardenafil	83	4.2	103	4.6	10	0.01	− 27	0.03
Zolpidem	87	6.7	112	20.6	− 7	0.05	− 34	0.07

Average relative recovery results were considered fully quantitative if within 80–120%, although compounds with lower accuracies were accepted as fully quantitative provided the average SD was < 20%. Semi-quantitative compounds again appear in italics. Briefly, 66 analytes in river water and 52 analytes in influent wastewater showed relative recovery at 80–120%. Twenty-nine compounds in river water and 43 compounds in influent wastewater had recoveries < 80% or > 120% (Table 2). Signal suppression should ideally be close to zero, with a negative signal suppression indicating signal enhancement of the analytes in matrix. The results are presented in Table 2. Most compounds were observed to have signal suppression of < 20% and signal enhancement of < 20%. Exceptions include oxymorphone and terbutaline with high signal suppression exceeding 80%, and ketoprofen and propylparaben with high signal enhancement exceeding 80% in river water. In influent wastewater, no analytes had signal suppression exceeding 80%, but eight analytes (benzophenone-1, candesartan cilexetil, diazinon, iopromide, ketoprofen, propylparaben, sotalol and triclosan) had high signal enhancement of > 80%.

Method recovery values were used to calculate method limits of detection and quantification, which are presented by matrix in Tables 3 and 4. Most analytes had mLOD of < 33 ng L^−1^ and mLOQ of < 78 ng L^−1^; exceptions include benzophenone-4, ethylparaben, ketoprofen and sotalol.

**Table 3:** Method limits of detection, quantification, method accuracy and method precision in river water (*n* = 9) (semi-quantitative compounds are presented in italics)

Analyte	River water					
	mLOD (μg L^−1^)	mLOQ (μg L^−1^)	Average accuracy		Average precision	
			%	SD	%	SD
Aminorex	0.009	0.02	113.4	34.8	3.5	2.4
Anhydroecgonine methylester	0.0004	0.0007	130.3	22.4	1.4	0.9
Benzophenone-1	0.01	0.06	110.8	25.5	9.2	0.6
Benzophenone-4	0.2	0.4	99.0	9.5	11.7	9.6
Benzoylecgonine	0.0001	0.0006	110.3	18.1	2.2	2.1
*Benzylpiperizine*	*0.004*	*0.02*	*89.8*	*2.4*	*3.6*	*2.9*
Bezafibrate	0.01	0.06	105.9	18.5	12.6	3.8
Buprenorphine	0.02	0.09	12.2	3.6	17.4	8.7
*Candesartan cilexetil*	*0.0002*	*0.001*	*2393.5*	*1226.7*	*3.5*	*0.7*
Carbamazepine	0.001	0.007	119.1	13.4	1.3	0.4
Carbamazepine 10,11 epoxide	0.001	0.006	106.1	16.6	4.6	3.6
*Carprofen*	*0.1*	*0.3*	*83.5*	*0.3*	*4.8*	*3.5*
Citalopram	0.002	0.008	112.3	42.3	3.1	1.3
Clothiniadin	0.0007	0.001	135.9	17.4	3.3	1.2
Cocaethylene	0.0006	0.001	122.8	21.7	3.2	0.6
Cocaine	0.0007	0.001	111.2	16.2	1.9	0.6
Codeine	0.0007	0.001	111.6	9.9	5.8	3.0
Cotinine	n/a	n/a	97.0	21.4	1.2	1.0
Desmethylcitalopram	0.02	0.08	1587.2	1012.6	0.7	0.4
*DHMA*	*0.1*	*0.2*	*108.8*	*1.5*	*21.2*	*24.0*
Diazepam	0.01	0.02	123.5	15.3	5.5	5.8
Diazinon	0.0007	0.001	663.3	226.3	4.4	1.6
Diclofenac	0.01	0.05	103.3	2.1	12.6	2.6
Dihydrocodeine	0.01	0.07	111.5	20.0	3.0	1.8
Dihydroketoprofen	0.1	0.3	81.7	13.6	20.9	2.3
Dihydromorphine	0.002	0.01	76.2	7.2	3.6	0.7
*Diltiazem*	*0.003*	*0.006*	*642.5*	*374.1*	*0.6*	*0.5*
*Duloxetine*	*0.0006*	*0.003*	*810.0*	*299.2*	*6.9*	*3.6*
E1-10,11-Dihydro-10-hydroxycarbamazepine	0.07	0.2	110.3	19.8	5.8	3.2
E1-Alprenolol	0.0007	0.003	87.4	18.1	5.7	2.5
E1-Atenolol	0.003	0.006	115.4	11.0	5.9	2.3
E1-Bisoprolol	0.0007	0.004	78.9	15.8	3.2	2.4
E1-Metoprolol	0.003	0.0006	78.8	13.9	3.0	0.3
E1-Mirtazapine	0.0001	0.007	124.3	19.0	2.6	1.1
E1-Oxazepam	0.0007	0.003	94.7	8.6	8.0	2.5
E1-Propanolol	0.007	0.01	87.2	14.2	4.3	2.9
*E1-Tramadol*	*0.003*	*0.006*	*95.1*	*18.5*	*5.4*	*1.5*
E2-10,11-Dihydro-10-hydroxycarbamazepine	0.006	0.01	122.5	17.6	5.2	4.5
E2-Alprenolol	0.0006	0.003	107.6	20.4	8.7	7.2
E2-Atenolol	0.003	0.006	115.6	13.9	5.4	3.1
E2-Bisoprolol	0.003	0.007	100.9	19.1	2.9	0.8
E2-Metoprolol	0.01	0.07	84.2	9.9	5.1	4.7
E2-Mirtazapine	0.0002	0.008	128.7	18.0	3.4	1.1
E2-Oxazepam	0.0005	0.001	87.5	17.5	6.2	1.5
E2-Propanolol	0.004	0.07	87.5	17.6	4.7	2.5
Ethylparaben	0.7	1	101.6	18.8	2.2	0.7
*Fexofenadine*	*0.2*	*0.4*	*62.8*	*9.8*	*9.0*	*2.6*
Griseofulvin	0.008	0.02	108.1	16.1	5.2	1.5
Heroin	0.002	0.009	110.8	27.4	4.3	1.1
HMA	0.02	0.03	110.7	14.6	4.9	3.7
*HMMA*	*0.0007*	*0.001*	*115.0*	*27.4*	*4.1*	*1.2*
Hydrocodone	0.0009	0.002	97.2	36.4	2.2	1.7
Imatinib	0.007	0.01	1695.3	601.5	1.3	0.4
Imidacloprid	0.002	0.003	74.3	16.7	2.3	0.8
Indoprofen	0.001	0.007	88.2	9.9	5.0	2.1
*Iopromide*	*0.002*	*0.009*	*222.9*	*114.9*	*3.2*	*2.5*
Ketamine	0.001	0.006	121.5	20.2	2.7	1.2
Ketoprofen	0.3	0.7	121.3	41.0	14.5	0.1
MDA	0.001	0.007	135.8	15.1	2.1	0.3
MDMA	0.001	0.006	87.1	15.6	2.0	0.3
MDPV	0.0001	0.0007	84.7	6.3	3.0	0.9
*Memantine*	*0.007*	*0.01*	*184.4*	*61.2*	*9.3*	*7.6*
Mephedrone	0.0001	0.0007	124.2	25.9	4.1	2.2
Metazachlor	0.01	0.07	107.6	28.6	2.8	1.1
Methadone	0.0006	0.001	100.1	10.8	1.7	0.3
Methamphetamine	0.0002	0.0009	150.0	15.7	2.2	0.3
Methylparaben	0.01	0.05	106.9	12.1	5.0	2.0
Morphine	0.001	0.005	104.9	16.7	8.1	7.3
Nordiazepam	0.005	0.03	115.2	15.9	2.0	1.3
Norephedrine	0.003	0.02	75.5	1.6	2.4	0.4
Normorphine	0.001	0.002	39.8	9.9	12.1	10.1
Nortriptyline	0.001	0.006	91.8	6.7	2.4	1.1
*O-Desmethylnaproxen*	*0.7*	*2*	*109.0*	*2.0*	*5.1*	*4.0*
Omeprazole	0.005	0.01	121.9	15.4	1.1	0.6
Oxadiazon	0.007	0.01	731.2	362.3	2.3	2.7
Oxycodone	0.06	0.1	106.1	22.3	2.4	0.9
*Oxymorphone*	*0.0003*	*0.002*	*1143.7*	*597.5*	*4.8*	*6.1*
Pholcodine	n/a	n/a	78.4	18.9	6.5	2.3
Praziquantrel	0.0001	0.0001	125.1	16.7	4.1	1.1
Propylparaben	0.05	0.1	111.0	25.6	7.4	0.9
Quetiapine	0.00004	0.0002	114.2	29.0	2.4	0.9
Risperidone	0.0008	0.002	426.6	191.5	1.4	0.4
Salbutamol	0.007	0.01	122.5	33.0	2.2	0.2
Sotalol	0.1	0.4	649.6	345.1	1.9	1.2
Sulphadiazine	0.001	0.002	109.8	4.5	4.7	3.1
Sulphamethoxazole	0.0005	0.001	69.1	11.5	6.0	1.7
Sulphapyridine	0.001	0.007	87.9	12.7	1.9	0.5
Terbutaline	0.001	0.002	− 15.4	15.3	− 0.5	0.2
Terbuthylazine	0.01	0.05	584.4	173.4	2.7	1.4
Tetramisole	0.006	0.01	119.1	20.0	4.4	1.6
*Thiamethoxam*	*0.000004*	*0.00002*	*61.8*	*10.3*	*3.9*	*1.7*
*Triclosan*	*0.05*	*0.1*	*103.5*	*2.6*	*7.6*	*5.6*
Valsartan	0.06	0.1	87.0	9.9	14.9	8.6
Vardenafil	0.0007	0.001	510.1	286.9	0.8	0.3
Zolpidem	0.3	0.8	82.4	12.9	11.8	12.3

**Table 4:** Method limits of detection, quantification, method accuracy and method precision in influent wastewater (*n* = 9) (semi-quantitative compounds are presented in italics)

Analyte	Wastewater					
	mLOD (μg L^−1^)	mLOQ (μg L^−1^)	Average accuracy		Average precision	
			%	SD	%	SD
Aminorex	0.005	0.009	105.3	22.6	2.4	2.0
Anhydroecgonine methylester	0.0001	0.0003	111.6	11.1	2.0	1.6
Benzophenone-1	0.01	0.07	66.0	9.8	14.2	8.8
Benzophenone-4	0.2	0.5	107.6	9.0	5.4	2.1
Benzoylecgonine	0.0001	0.0005	101.2	18.6	1.1	0.4
*Benzylpiperizine*	*0.002*	*0.008*	*74.5*	*8.5*	*3.4*	*3.0*
Bezafibrate	0.01	0.07	99.9	8.9	11.5	6.3
Buprenorphine	0.006	0.03	10.0	0.7	8.0	5.2
*Candesartan cilexetil*	*0.0001*	*0.0005*	*1398.0*	*610.8*	*4.8*	*1.6*
Carbamazepine	0.001	0.006	120.6	13.9	0.7	0.2
Carbamazepine 10,11 epoxide	0.001	0.005	104.0	22.3	2.1	1.0
*Carprofen*	*0.2*	*0.5*	*116.9*	*25.9*	*9.5*	*1.7*
Citalopram	0.001	0.006	203.4	63.2	2.5	0.4
Clothiniadin	0.0006	0.001	101.0	9.9	2.7	0.3
Cocaethylene	0.0006	0.001	120.4	14.1	1.7	0.8
Cocaine	0.0006	0.001	118.7	17.5	1.2	0.5
Codeine	0.0005	0.001	169.9	52.4	3.2	1.6
Cotinine	0.007	0.04	49.3	146.7	1.6	0.5
Desmethylcitalopram	0.01	0.06	907.1	554.9	1.6	0.0
*DHMA*	*0.1*	*0.2*	*117.6*	*17.3*	*3.9*	*3.0*
Diazepam	0.006	0.01	110.3	9.8	5.2	1.8
Diazinon	0.0007	0.001	336.4	80.7	5.2	2.4
Diclofenac	0.01	0.05	132.3	23.4	7.0	6.3
Dihydrocodeine	0.02	0.08	128.9	20.4	2.2	1.1
Dihydroketoprofen	0.2	0.5	135.7	46.8	7.5	2.8
Dihydromorphine	0.003	0.02	80.4	13.9	4.0	1.9
*Diltiazem*	*0.001*	*0.002*	*1007.7*	*634.0*	*1.6*	*0.5*
*Duloxetine*	*0.0006*	*0.003*	*387.4*	*98.1*	*5.4*	*1.8*
E1-10,11-Dihydro-10-hydroxycarbamazepine	0.04	0.1	109.0	18.8	1.3	0.3
E1-Alprenolol	0.0008	0.004	107.8	12.7	2.4	1.1
E1-Atenolol	0.003	0.006	116.2	14.1	1.1	0.2
E1-Bisoprolol	0.0006	0.003	99.4	20.7	2.3	1.6
E1-Metoprolol	0.003	0.0006	85.9	11.8	1.3	0.3
E1-Mirtazapine	0.0001	0.006	220.5	120.8	4.2	2.8
E1-Oxazepam	0.0006	0.003	103.1	4.1	8.0	2.2
E1-Propanolol	0.005	0.01	90.7	13.3	2.0	1.2
*E1-Tramadol*	*0.007*	*0.01*	*165.5*	*64.8*	*4.6*	*0.7*
E2-10,11-Dihydro-10-hydroxycarbamazepine	0.005	0.01	109.0	13.8	3.3	0.7
E2-Alprenolol	0.0006	0.003	100.7	9.5	3.9	2.4
E2-Atenolol	0.003	0.006	112.8	9.4	1.2	0.8
E2-Bisoprolol	0.003	0.005	99.8	20.3	1.3	0.8
E2-Metoprolol	0.01	0.06	116.1	13.6	4.5	4.5
E2-Mirtazapine	0.0001	0.007	164.2	36.2	4.6	4.2
E2-Oxazepam	0.001	0.002	104.0	11.7	5.8	3.0
E2-Propanolol	0.003	0.05	133.1	16.8	1.4	1.0
Ethylparaben	0.6	1	122.0	16.3	4.5	2.9
*Fexofenadine*	*0.3*	*0.6*	*133.2*	*39.8*	*9.1*	*1.1*
Griseofulvin	0.008	0.02	91.8	14.8	3.4	1.5
Heroin	0.001	0.006	125.5	10.5	4.8	0.8
HMA	0.008	0.02	83.6	4.4	3.0	1.3
*HMMA*	*0.0004*	*0.0008*	*69.9*	*7.5*	*2.3*	*2.5*
Hydrocodone	0.0008	0.002	105.7	15.7	2.1	0.8
Imatinib	0.004	0.008	1191.8	445.9	1.2	0.6
Imidacloprid	0.001	0.003	56.0	7.4	3.2	2.1
Indoprofen	0.002	0.008	99.1	15.6	3.9	3.4
*Iopromide*	*0.001*	*0.006*	*148.0*	*20.8*	*8.7*	*5.5*
Ketamine	0.001	0.006	104.7	12.0	2.1	0.8
Ketoprofen	0.4	0.9	110.7	2.9	4.0	3.2
MDA	0.001	0.006	113.4	34.6	1.5	0.2
MDMA	0.001	0.006	85.9	16.9	1.2	0.3
MDPV	0.0001	0.0007	116.1	13.3	2.0	0.6
*Memantine*	*0.006*	*0.01*	*82.9*	*17.4*	*4.4*	*0.5*
Mephedrone	0.0002	0.0009	148.7	21.7	0.8	0.5
Metazachlor	0.008	0.04	122.9	15.2	5.2	4.1
Methadone	0.0007	0.001	143.7	25.0	2.6	0.5
Methamphetamine	0.0001	0.0007	118.7	31.3	2.1	0.5
Methylparaben	0.01	0.05	103.5	9.2	2.6	0.3
Morphine	0.001	0.005	31.7	107.8	2.5	1.4
Nordiazepam	0.002	0.009	100.6	14.4	3.0	1.6
Norephedrine	0.002	0.009	90.2	2.8	2.6	0.5
Normorphine	0.0003	0.0006	42.1	14.7	2.1	0.3
Nortriptyline	0.001	0.006	82.0	10.2	5.3	1.6
*O-Desmethylnaproxen*	*0.2*	*0.6*	*98.7*	*26.0*	*6.4*	*3.0*
Omeprazole	0.01	0.03	89.2	14.5	2.1	0.7
Oxadiazon	0.007	0.01	633.6	308.4	6.2	0.7
Oxycodone	0.008	0.02	97.2	32.0	8.6	10.1
*Oxymorphone*	*0.0002*	*0.0009*	*303.9*	*93.7*	*5.3*	*1.2*
Pholcodine	0.006	0.03	50.7	13.3	10.0	7.1
Praziquantrel	0.0001	0.0001	110.7	15.3	5.6	1.0
Propylparaben	0.08	0.2	124.9	13.1	2.0	2.3
Quetiapine	0.00001	0.00006	96.5	36.8	2.3	0.3
Risperidone	0.0007	0.001	361.9	153.9	1.3	0.4
Salbutamol	0.007	0.01	96.7	20.3	1.0	0.5
Sotalol	0.3	0.7	421.5	114.2	2.5	1.6
Sulphadiazine	0.0007	0.001	88.7	6.9	3.5	0.8
Sulphamethoxazole	0.0005	0.0009	67.3	3.4	2.1	0.3
Sulphapyridine	0.0009	0.005	128.4	2.7	1.4	0.2
Terbutaline	0.001	0.002	− 17.9	17.7	− 1.0	0.7
Terbuthylazine	0.01	0.05	506.5	137.4	1.3	0.6
Tetramisole	0.007	0.01	121.8	11.8	7.1	6.5
*Thiamethoxam*	*0.000003*	*0.00001*	*47.1*	*8.8*	*6.7*	*4.1*
*Triclosan*	*0.08*	*0.2*	*170.8*	*125.2*	*2.8*	*3.1*
Valsartan	0.08	0.2	82.1	2.8	5.0	3.9
Vardenafil	0.0005	0.0009	425.1	166.4	1.8	0.4
Zolpidem	0.06	0.2	64.2	4.7	13.8	11.2

Semi-quantitative compounds again appear in italics. Likewise, precision results should be as close to zero as possible and should be < 20% RSD. Most analytes performed well with accuracies between 80 and 120% and precision of < 20% (Tables 3 and 4). Exceptions include candesartan cilexetil, diltiazem, duloxetine, fexofenadine and thiamethoxam. The only analytes with method precision of > 20% were DHMA and dihydroketoprofen in river water only, with an average precision of 21%.

Resolution of chiral compounds and enantiomeric fractions were calculated at three concentrations, and the average results are presented in Table 5. Resolution was excellent in both matrices, although generally better in river water, due to narrower peak widths and greater *S*/*N* from a ‘cleaner’ matrix. 10,11-Dihydro-10-hydroxycarbamazepine had better resolution in wastewater than river water as the E1-enantiomer did not always have a quantifiable signal to noise (*S*/*N*) ratio in the latter matrix. This, coupled with a narrow peak width, led to relatively greater separation of the two enantiomers in wastewater, rather than in river water where both were detected with a quantifiable *S*/*N* and a broader peak width.

**Table 5:** Method resolution of enantiomers and enantiomeric fractions (*n* = 9)

Analyte	River water				Wastewater			
	*R*_*s*_	SD	EF	SD	*R*_*s*_	SD	EF	SD
10,11-Dihydro-10-hydroxycarbamazepine	13.42	2.34	0.45	0.07	28.56	22.11	0.41	0.09
Alprenolol	21.92	4.73	0.52	0.01	14.90	2.52	0.45	0.05
Atenolol	20.84	4.26	0.48	0.02	9.48	5.80	0.50	0.02
Bisoprolol	46.42	30.58	0.53	0.01	24.82	8.03	0.54	0.02
Metoprolol	50.22	44.11	0.52	0.02	14.58	2.81	0.50	0.01
Mirtazapine	7.07	2.02	0.51	0.01	6.43	3.17	0.52	0.01
Oxazepam	6.54	2.01	0.53	0.03	13.89	11.71	0.45	0.13
Propanolol	46.41	36.90	0.42	0.11	14.32	4.89	0.47	0.04
Tramadol	3.76	1.30	–	–	5.08	1.35	–	–

In summary, after validation, out of 140 analytes tested, there were eighty-one compounds where fully quantitative information could be determined, and fourteen semi-quantitatively analysed compounds: benzylpiperazine, candesartan cilexetil, carprofen, DHMA, diltiazem, duloxetine, E1-tramadol, fexofenadine, HMMA, iopromide, memantine, oxymorphone, thiamethoxam and triclosan. There was no clear difference between pKa and Log P of the fully and semi-quantitative analytes, although the semi-quantitative analytes had a slightly higher average log P (3.4 ± 2.5 vs 2.4 ± 1.6 respectively). Likewise, there was no significant difference between the Log P and pKa of the 45 qualitative analytes and the 95 quantitative or semi-quantitative analytes. Several of these qualitative analytes performed poorly with very low or very high average relative recoveries, despite using a deuterated analogue of the analyte as the internal standard and good instrument performance results.

The ninety-five semi- or fully quantitative compounds included analytes from a range of environmentally important classes including seven herbicides, insecticides and pesticides, which enter the environment directly (without wastewater treatment) as run-off from agriculture, as well as five antifungal compounds, which are routinely added to personal care products. Additionally, carprofen and sulphapyridine are licensed for veterinary use and may also enter the environment directly. Most of the other analytes are primarily classified as human pharmaceuticals including, antidepressants, beta-blockers, non-steroidal anti-inflammatory drugs (NSAIDs) and opioids. In particular, the beta-blockers performed very well in this method and were all fully quantitative, which was expected as the SFC method was selected because it effectively separated beta-blockers. Monitoring these pharmaceuticals is important for assessing both public health, via influent wastewater, and environmental health, via effluent wastewater and river water, particularly considering European directives concerning water quality and reducing the environmental impact of human pharmaceutical usage (European Parliament & Council, 2002, Commission, 2019). The remaining fourteen analytes are primarily classified as drugs of abuse or their metabolites, although ketamine is also widely used in veterinary medicine and so may also enter the environment directly. Whilst these compounds are primarily of interest for monitoring drug consumption within human populations, they are also analogous to other pharmaceuticals as potential compounds of environmental concern.

### Environmental analysis

Environmental samples were analysed using the validated method. Average concentrations in each matrix are recorded in Table 6. The average enantiomeric fraction and average peak resolution for chiral analytes in each matrix are presented in Table 7. CECs were quantified in river water at concentrations spanning from < LOQ (aminorex, AEME, benzophenone-1, benzylpiperizine, candesartan cilexetil, carbamazepine-10,11-epoxide, carprofen, cocaethylene, cocaine, DHMA, diazepam, diclofenac, dihydroketoprofen, dihydromorphine, E1 & E2-10,11-hydrodyo-10-hydroxycarbamazepine, E1 & E2-alprenolol, E1 & E2-metoprolol, E1-propanolol, fexodenadine, HMA, HMMA, indoprofen, iopromide, ketoprofen, MDPV, memantine, mephedrone, norephedrine, O-desmethylnaproxen, oxycodone, oxymorphone, pholcodine, sulphadiazine, sulphamethoxazole, tetramisole, thiamethoxam and valsartan) through 0–988 ng L^−1^ (benzoylecgonine, buprenorphine, carbamazepine, citalopram, clothiniadin, codeine, cotinine, desmethylcitalopram, diazinon, dihydrocodeine, diltiazem, duloxetine, E1 & E2-atenolol, E1 & E2-mirtazapine, E1 & E2-oxazepam, E1-tramadol, E2-bisoprolol, E2-propanolol, ethylparaben, griseofulvin, heroin, hydrocodone, imatinib, imidacloprid, ketamine, MDA, MDMA, metazachlor, methadone, methamphetamine, methylparaben, morphine, nordiazepam, normorphine, nortriptyline, omeprazole, oxadiazon, praziquantrel, propylparaben, quetiapine, salbutamol, sulphapyridine, terbuthylazine and zolpidem) to 1–106 μg L^−1^ (benzophenone-4, risperidone, sotalol, terbutaline, triclosan and vardenafil). Interestingly, detected concentrations of some CECs were lower in wastewater influent (20–26,923 μg L^−1^, average concentration 7402 μg L^−1^) than in effluent wastewater (50–136,080 μg L^−1^, average concentration 8260 μg L^−1^), which may reflect influence from microbial metabolic processes during wastewater treatment. For example, a metabolite of citalopram (desmethylcitalopram) was found at concentrations of 1382 μg L^−1^ in wastewater influent and at 1693 μg L^−1^ in wastewater effluent. Likewise, oxazepam had greater concentrations in effluent wastewater (1732 μg L^−1^) than in influent wastewater (952 μg L^−1^) which could result from it being a common metabolite of several other benzodiazepines, such as diazepam. However, as the water used in this experiment was collected by grab sampling, it was not possible to conclusively say that this observation was due to metabolic processes occurring during wastewater treatment. Similarly, some analytes, such as oxadiazon and terbuthylazine, had a greater concentration in river water than in influent or effluent wastewater. This is likely because they are used as pesticides and so are entering the environment directly, e.g. through runoff from fields and gardens, rather than from human consumption.

**Table 6:** Analysis of environmental samples in river water, effluent and influent wastewater (semi-quantitative compounds are presented in italics)

Analyte	River water		Effluent		Influent	
	Average concentration (ng L^−1^) (*n* = 6)	SD	Average concentration (ng L^−1^) (*n* = 6)	SD	Average concentration(ng L^−1^) (*n* = 6)	SD
Aminorex	< LOQ	< LOQ	< LOQ	< LOQ	< LOQ	< LOQ
Anhydroecgonine methylester	< LOQ	< LOQ	2873.3	42.5	2371.7	25.1
Benzophenone-1	< LOQ	< LOQ	< LOQ	< LOQ	1211.7	24.0
Benzophenone-4	2101.7	20.7	60,825.0	6.5	18,363.3	10.9
Benzoylecgonine	538.3	4.1	7998.3	1.4	22,773.3	2.2
*Benzylpiperizine*	< *LOQ*	< *LOQ*	< *LOQ*	< *LOQ*	*217.5*	*5.4*
Bezafibrate	< LOQ	< LOQ	9781.7	42.7	8821.7	21.3
Buprenorphine	290.8	14.3	295.0	18.8	307.5	17.4
*Candesartan cilexetil*	< *LOQ*	< *LOQ*	< *LOQ*	< *LOQ*	< *LOQ*	< *LOQ*
Carbamazepine	311.7	6.3	5325	1.6	4191.7	1.4
Carbamazepine 10,11 epoxide	< LOQ	< LOQ	< LOQ	< LOQ	237.5	34.9
Carprofen	< LOQ	< LOQ	< LOQ	< LOQ	< LOQ	< LOQ
Citalopram	826.7	3.6	6176.7	3.6	5083.3	1.8
Clothiniadin	486.7	5.1	168.3	16.2	150.0	14.4
Cocaethylene	< LOQ	< LOQ	< LOQ	< LOQ	285.0	7.8
Cocaine	< LOQ	< LOQ	1698.3	2.5	9338.3	2.7
Codeine	988.3	6.7	24,883.3	3.7	20,900.0	3.2
Cotinine	260.0	4.4	6206.67	0.5	15,716.7	2.3
Desmethylcitalopram	335.0	6.6	1693.3	5.9	1381.7	5.2
*DHMA*	< *LOQ*	< *LOQ*	< *LOQ*	< *LOQ*	< *LOQ*	< *LOQ*
Diazepam	< LOQ	< LOQ	50.0	54.2	20.0	70.7
Diazinon	242.5	29.7	< LOQ	< LOQ	161.7	12.6
Diclofenac	< LOQ	< LOQ	8988.3	40.4	1648.4	20.9
Dihydrocodeine	236.7	7.2	4298.3	11.2	2951.7	4.8
Dihydroketoprofen	< LOQ	< LOQ	< LOQ	< LOQ	< LOQ	< LOQ
Dihydromorphine	< LOQ	< LOQ	511.7	4.8	538.3	5.8
*Diltiazem*	*266.7*	*1.8*	*1033.3*	*3.9*	*1083.3*	*3.7*
*Duloxetine*	*130.0*	*6.3*	*236.7*	*9.3*	*253.3*	*15.6*
E1-10,11-Dihydro-10-hydroxycarbamazepine	< LOQ	< LOQ	< LOQ	< LOQ	< LOQ	< LOQ
E1-Alprenolol	< LOQ	< LOQ	< LOQ	< LOQ	< LOQ	< LOQ
E1-Atenolol	103.33	40.5	4468.33	2.4	6211.7	5.2
E1-Bisoprolol	< LOQ	< LOQ	791.67	9.4	808.33	5.3
E1-Metoprolol	< LOQ	< LOQ	61.7	49	65.0	34.1
E1-Mirtazapine	71.67	9.6	1003.3	3.8	621.7	5.4
E1-Oxazepam	118.3	37	856.7	18.1	396.7	18.6
E1-Propanolol	< LOQ	< LOQ	1170.0	6.2	916.7	9.4
*E1-Tramadol*	*401.7*	*6.8*	*3691.7*	*9*	*2348.3*	*3.1*
E2-10,11-Dihydro-10-hydroxycarbamazepine	< LOQ	< LOQ	2086.7	11.8	1031.7	8.8
E2-Alprenolol	< LOQ	< LOQ	< LOQ	< LOQ	< LOQ	< LOQ
E2-Atenolol	193.3	12.2	4583.3	5.4	5925	4.5
E2-Bisoprolol	113.3	4.2	1086.7	2.2	1003.3	4.6
E2-Metoprolol	< LOQ	< LOQ	< LOQ	< LOQ	< LOQ	< LOQ
E2-Mirtazapine	96.7	7.7	446.7	5.4	295.0	6.4
E2-Oxazepam	241.7	12	875.0	18.7	555	18
E2-Propanolol	245.0	8.7	1970	5.8	1436.7	4.5
Ethylparaben	511.7	2.6	548.3	6.3	2376.7	9.1
*Fexofenadine*	< *LOQ*	< *LOQ*	*27,843.3*	*50.7*	*10,281.7*	*21.8*
Griseofulvin	150.0	19.6	157.5	13.7	205	22.6
Heroin	305.0	5.3	< LOQ	< LOQ	343.33	15.4
HMA	< LOQ	< LOQ	< LOQ	< LOQ	< LOQ	< LOQ
HMMA	< LOQ	< LOQ	346.7	16.4	16.7	44.9
Hydrocodone	791.7	4.7	7540.0	2.3	5551.7	1.8
Imatinib	183.3	10.3	301.7	14.8	368.3	12.1
Imidacloprid	446.7	6.2	3091.7	5.8	788.3	7.3
Indoprofen	< LOQ	< LOQ	< LOQ	< LOQ	< LOQ	< LOQ
*Iopromide*	< *LOQ*	< *LOQ*	*14,861.7*	*35.3*	< *LOQ*	< *LOQ*
Ketamine	148.3	7.2	3026.7	5.5	2371.7	3.8
Ketoprofen	< LOQ	< LOQ	< LOQ	< LOQ	< LOQ	< LOQ
MDA	216.7	5.8	870.0	4.3	476.7	3.8
MDMA	31.7	11.8	2458.3	1.8	3945.0	2.2
MDPV	< LOQ	< LOQ	< LOQ	< LOQ	< LOQ	< LOQ
*Memantine*	< *LOQ*	< *LOQ*	*391.7*	*27.7*	*226.7*	*9.8*
Mephedrone	< LOQ	< LOQ	< LOQ	< LOQ	< LOQ	< LOQ
Metazachlor	299.7	3.9	278.3	5.3	276.7	1.7
Methadone	10.0	0.0	431.7	3.4	513.3	2.7
Methamphetamine	141.7	2.6	263.3	3.6	268.3	5.5
Methylparaben	371.7	27.9	440	23.2	16,301.7	12
Morphine	328.3	5.4	8661.7	6.3	11,210.0	2.7
Nordiazepam	55.0	9.1	230.0	14.6	171.7	14
Norephedrine	< LOQ	< LOQ	< LOQ	< LOQ	< LOQ	< LOQ
Normorphine	731.67	2.1	1438.3	18.4	1810.0	9.6
Nortriptyline	563.3	10.9	923.3	5.1	616.7	7.8
O-Desmethy lnaproxen	< LOQ	< LOQ	25,875.0	47.6	35,981.7	21.3
Omeprazole	321.7	2.1	508.3	4.5	1468.3	3.3
Oxadiazon	536.7	30.6	< LOQ	< LOQ	< LOQ	< LOQ
Oxycodone	< LOQ	< LOQ	373.33	20.5	700	0.0
*Oxymorphone*	< *LOQ*	< *LOQ*	*455.0*	*19.5*	*435.0*	*13.6*
Pholcodine	< LOQ	< LOQ	44,582.5	36.8	26,923.3	44.2
Praziquantrel	15.0	50.9	90.0	9.1	28.3	51.6
Propylparaben	550.0	1.8	611.7	7.3	3583.3	7.6
Quetiapine	346.7	1.4	506.7	3.2	751.7	1.8
Risperidone	3683.3	16.5	4286.7	16.8	2440	13.8
Salbutamol	238.3	98.0	413.3	60.7	8078.0	215.4
Sotalol	6198.3	32.6	97,220	9.3	44,135.0	10.5
Sulphadiazine	< LOQ	< LOQ	288.3	38.7	< LOQ	< LOQ
Sulphamethoxazole	< LOQ	< LOQ	6426.7	9.0	2590.0	11.5
Sulphapyridine	496.7	5.5	18,958.3	13.7	13,751.7	4.5
Terbutaline	106,647.0	17.9	136,080.0	14.4	206,545.0	12.7
Terbuthylazine	126.7	5.9	< LOQ	< LOQ	83.3	6.0
Tetramisole	< LOQ	< LOQ	233.3	9.5	256.7	15.0
*Thiamethoxam*	< *LOQ*	< *LOQ*	< *LOQ*	< *LOQ*	< *LOQ*	< *LOQ*
*Triclosan*	*1325.0*	*9.4*	*1890.0*	*44.4*	*6185.0*	*31.0*
Valsartan	< LOQ	< LOQ	5481.7	47.0	2325.0	20.9
Vardenafil	2618.3	27.5	1691.7	33.5	1201.7	46.3
Zolpidem	190.0	0.4	240.0	10.6	< LOQ	< LOQ

**Table 7:** Average enantiomeric fraction and separation of chiral analytes in matrix ± standard deviation (*n* = 9)

Analytes	River water		Effluent		Influent	
	EF	*R*_*s*_	EF	*R*_*s*_	EF	*R*_*s*_
10,11-Dihydro-10-hydroxycarbamazepine	< LOQ	18.2 ± 1.4	0.00 ± 0.01	14.6 ± 1.1	0.0 ± 0.01	14.5 ± 0.9
Alprenolol	< LOQ	5.4 ± 1.1	< LOQ	5.5 ± 0.4	< LOQ	5.3 ± 0.6
Atenolol	0.30 ± 0.10	30.5 ± 2.4	0.5 ± 0.01	30.3 ± 1.2	0.5 ± 0.01	32.4 ± 1.3
Bisoprolol	0.00 ± 0.01	10.1 ± 1.8	0.4 ± 0.01	7.9 ± 0.4	0.4 ± 0.01	7.6 ± 0.4
Metoprolol	< LOQ	< LOQ	1.0 ± 0.01	12.2 ± 4.7	1.0 ± 0.01	10.5 ± 0.9
Mirtazapine	0.40 ± 0.01	8.2 ± 0.8	0.7 ± 0.01	6.7 ± 0.2	0.7 ± 0.01	8.6 ± 0.7
Oxazepam	0.30 ± 0.10	33.2 ± 5.0	0.5 ± 0.10	19.7 ± 2.1	0.4 ± 0.10	25.1 ± 2.7
Propanolol	< LOQ	22.4 ± 2.8	0.4 ± 0.01	23.2 ± 1.3	0.4 ± 0.01	21.8 ± 1.2
Tramadol	–	6.0 ± 0.3	–	6.0 ± 0.1	–	5.9 ± 0.2

Chiral CECs that were enantiomerically separated are presented in Table 7. Most analytes showed non-racemic EFs, which indicates enantiomer selective processes occurring due to either human metabolism or microbial processes. This in turn highlights the importance of understanding chirality for determining biological effects, including toxicity. For example, bisoprolol was the only beta-blocker quantified in all three matrices and was also enriched with the E2 isomer in river water compared with influent and effluent wastewater. The EF of mirtazapine appeared to vary considerably between wastewater influent and effluent, and river water, which suggested it was being preferentially metabolised favouring the E1 enantiomer in humans and the E2 enantiomer in the environment. To the authors’ knowledge, there is no literature data on the ecotoxicity of mirtazapine, although other antidepressants have been studied [46, 47]. The EF of oxazepam also varied between the matrices; however, the difference in EF was much less pronounced.

## Conclusion

The development of new analytical methods for the analysis of environmental micropollutants is important, particularly where critical information on chirality can be collected. SFC is an excellent technique for combined achiral-chiral analysis as it allows for the development of robust methods with shorter run times than would usually be achieved in chiral HPLC methods. This is due to the combined use of supercritical CO_2_, non-biological chiral selectors and smaller-UHPLC-size particles. The method development data shown highlighted the range of available SFC column chemistries and optimised chromatographic conditions for the development of new, combined non-chiral and chiral-SFC methods capable of separating a range of different chiral and non-chiral analytes. The final method showcases the power of SFC for the rapid analysis (within < 10 min) of chiral and achiral compounds in important environmental matrices. Whilst this method was only able to chirally separate nine analytes, the initial method development showed that under different chromatographic conditions, the same column could partially or fully separate another five analytes, with others separated under the same chromatographic conditions using alternative columns. In summary, out of 140 analytes selected for the study, 81 were fully quantifiable and validated, and 14 were semi-quantitative. mLOQs spanned 10 pg L^−1^–2 μg L^−1^ and accuracy and precision were maintained at 103 ± 11.1% and 4 ± 2.1% respectively. The analysis of environmental samples showed omnipresence of selected CECs, some showing enantioselective fate, such as mirtazapine. Overall, the CEL-1 methods gave excellent separation of chiral enantiomers and rapid quantitative analysis of 95 CEC, at the cost of reduced instrument and method sensitivity compared to contemporary achiral methodologies. However, these achiral methodologies also provide a road map for how to improve sensitivity without sacrificing the efficiency of SFC or focusing on only a handful of analytes.

## Electronic supplementary material

ESM 1 (PDF 3615 kb).
