# Supplementary material for: A multi-residue method by supercritical fluid chromatography coupled with tandem mass spectrometry method for the analysis of chiral and non-chiral chemicals of emerging concern in environmental samples
Source: Anal Bioanal Chem. 2020 Jul 9;412(23):5563–81. doi: 10.1007/s00216-020-02780-9 (PMC7413908; doi:10.1007/s00216-020-02780-9)
Supplement: Supplementary file 1 — (PDF 3615 kb). [file 216_2020_2780_MOESM1_ESM.pdf]

Analytical and Bioanalytical Chemistry

Electronic Supplementary Material

A multi-residue method by supercritical fluid chromatography coupled with tandem mass spectrometry method for the analysis of chiral and non-chiral chemicals of emerging concern in environmental samples

Jack Rice, Anneke Lubben, Barbara Kasprzyk-Hordern

Table S1 Supplier information and CAS numbers for all analytes and internal standards used in this paper

Compound	CAS number	Supplier
**1,7-dimethylxantine**	611-59-6	Sigma Aldrich
**1S,2R-(+)-ephedrine D3**	285979-73-9	LGC standards (Middlesex, UK)
**2-Hydroxyibuprofen**	51146-55-5	Sigma Aldrich (Gillingham, UK)
**2-Phenylproponic acid**	492-37-5	Sigma-Aldrich (Gillingham, UK)
**8-isoF2B**	177020-26-7	Cayman Chemicals
**Acetaminophen**	103-90-2	Sigma Aldrich
**Acetaminophen D4**	64315-36-2	Sigma Aldrich (Gillingham, UK)
**AEME**	43021-26-7	Sigma Aldrich (Cerilliant product)
**Aminorex**	2207-50-3	Sigma Aldrich (Gillingham, UK)
**Amoxicillin**	26787-78-0	Sigma Aldrich
**Amphetamine**	300-62-9	LGC (Cerilliant product)
**Amphetamine D5**	136765-27-0	LGC standards (Middlesex, UK)
**Ampicillin**	69-53-4	Sigma Aldrich
**Azathioprine**	446-86-6	Sigma Aldrich
**Azithromycin**	83905-01-5	Sigma Aldrich
**Benzophenone-1**	131-56-6	Sigma Aldrich
**Benzophenone-2**	131-55-5	Sigma Aldrich
**Benzophenone-3**	131-57-7	Sigma Aldrich
**Benzophenone-4**	4065-45-6	Sigma Aldrich
**Benzoylecgonine**	519-09-5	Sigma Aldrich
**Benzoylecgonine D8**	205446-21-5	LGC standards (Middlesex, UK)
**Benzylpiperizine**	2759-28-6	LGC
**Bezafibrate**	41859-67-0	Sigma Aldrich
**Bicalutamide**	90357-06-5	Sigma Aldrich
**Buprenorphine**	52485-79-7	Sigma Aldrich
**Caffeine**	58-08-2	Sigma Aldrich
**Candesartan cilexetil**	145040-37-5	Sigma Aldrich
**Capecitabine**	154361-50-9	Sigma Aldrich
**Carbamazepine**	298-46-4	Sigma Aldrich
**Carbamazepine-10,11-epoxide**	36507-30-9	LGC
**Carbamazepine 13C6**	298-46-4	Sigma Aldrich (Gillingham, UK)
**Carboxyibuprofen**	15935-54-3	Sigma Aldrich (Gillingham, UK)
**Carprofen**	53716-49-7	Sigma Aldrich (Gillingham, UK)
**Cetirizine**	83881-51-0	LGC
**Chloramphenicol**	56-75-7	Sigma Aldrich
**Chlorpyrifos**	2921-88-2	LGC (Dr Ehrenstorfer)
**Cimetidine**	51481-61-9	Sigma Aldrich
**Citalopram**	59729-33-8	Sigma Aldrich (Gillingham, UK)
**Citalopram D6**	1190003-26-9	Toronto Research Chemicals Inc. (Ontario, Canada)
**Clarithromycin**	81103-11-9	Sigma Aldrich
**Clothiniadin**	210880-92-5	Sigma Aldrich (PESTANAL)
**Cocaethylene**	529-38-4	Sigma Aldrich (Cerilliant product)
**Cocaethylene D3**	136765-30-5	LGC standards (Middlesex, UK)
**Cocaine**	50-36-2	LGC (Cerilliant product)
**Cocaine D3**	65266-73-1	LGC standards (Middlesex, UK)
**Codeine**	76-57-3	Sigma Aldrich
**Codeine D6**	1007844-34-9	LGC standards (Middlesex, UK)
**Cotinine**	486-56-6	Sigma Aldrich (Cerilliant product)
**Cotinine D3**	110952-70-0	LGC standards (Middlesex, UK)
**Creatinine**	60-27-5	Sigma Aldrich
**Cytarabine**	147-94-4	Sigma Aldrich
**Danofloxacin**	112398-08-0	Sigma Aldrich
**Desmethylcitalopram**	62498-67-3	Toronto Research Chemicals Inc. (Ontario, Canada)
**Desmethylvenlafaxine**	93413-62-8	Sigma Aldrich (Gillingham, UK)
**Desvenlafaxine**	300827-87-6	Sigma Aldrich
**DHMA**	15398-87-5	Kinesis
**Diazepam**	439-14-5	Sigma Aldrich (Cerilliant product)
**Diazepam D5**	65854-76-4	Toronto Research Chemicals Inc. (Ontario, Canada)
**Diazinon**	333-41-5	Sigma Aldrich (PESTANAL)
**Dichlofluanid**	1085-98-9	Sigma Aldrich (PESTANAL)
**Diclofenac**	15307-86-5	Sigma Aldrich
**Dihydrocodeine**	125-28-0	Sigma Aldrich
**Dihydro ketoprofen**	55453-87-7	Toronto Research Chemicals Inc. (Ontario, Canada)
**Dihydromorphine**	509-60-4	Sigma Aldrich (Cerilliant product)
**Diltiazem**	42399-41-7	Sigma Aldrich
**Donepezil**	120014-06-4	LGC
**Duloxetine**	116539-59-4	LGC
**E1 & E2-10,11-dihydro-10-hydroxycarbamazepine**	29331-92-8	LGC
**E1 & E2-Alprenolol**	13707-88-5	Sigma Aldrich (Gillingham, UK)
**E1 & E2-Atenolol**	29122-68-7	Sigma Aldrich (Gillingham, UK)

**E1 & E2-Atenolol D7**	1202864-50-3	Sigma Aldrich (Gillingham, UK)
**E1 & E2-Bisoprolol**	66722-44-9	Sigma Aldrich
**E1 & E2-Metoprolol**	51384-51-1	Sigma Aldrich (Gillingham, UK)
**E1 & E2-Mirtazapine**	85650-52-8	Sigma Aldrich
**E1 & E2-Mirtazapine D3**	1216678-68-0	Toronto Research Chemicals Inc. (Ontario, Canada)
**E1 & E2-Propanolol**	525-66-6	Sigma Aldrich
**E1 & E2-Tramadol**	27203-92-5	Sigma Aldrich (Gillingham, UK)
**E1& E2-Metoprolol D7**	12929006-91-2	Toronto Research Chemicals Inc. (Ontario, Canada)
**E1& E2-Oxazepam**	604-75-1	Sigma Aldrich (Cerilliant product)
**E1& E2-Oxazepam D5**	65854-78-6	Sigma Aldrich (Cerilliant product)
**E1& E2-Propanolol D7**	344298-99-3	Sigma Aldrich (Gillingham, UK)
**Ecgonine methyl ester D3**	136765-34-9	Sigma Aldrich (Cerilliant product)
**EE2**	57-63-6	Sigma Aldrich
**Ephedrine**	50-98-6	Sigma Aldrich
**Erythromycin**	114-07-8	Sigma Aldrich
**Ethylparaben**	120-47-8	Sigma Aldrich
**Fexofenadine**	83799-24-0	LGC
**Flufenacet**	142459-58-3	LGC (Dr Ehrenstorfer)
**Flumequine**	42835-25-6	Sigma Aldrich
**Fluoxetine**	54910-89-3	LGC (Cerilliant product)
**Fluoxetine D5**	1173020-43-3	Toronto Research Chemicals Inc. (Ontario, Canada)
**Furosemide**	54-31-9	Sigma Aldrich
**Gabapentin**	60142-96-3	LGC (Cerilliant product)
**Gabapentin D4**	1185039-20-6	TRC (Toronto Research Chemicals, Toronto, Canada)
**Gemfibrozil**	25812-30-0	Sigma Aldrich
**Gliclazide**	21187-98-4	LGC
**Griseofulvin**	126-07-8	Sigma Aldrich
**Heroin**	561-27-3	Sigma Aldrich (Cerilliant product)
**Heroin D9**	1338713-49-7	LGC standards (Middlesex, UK)
**HMA**	13062-61-8	Kinesis
**HMMA**	438625-58-2	Kinesis
**HNE-MA**	75899-68-2	Cayman Chemicals
**Hydrocodone**	125-29-1	Sigma Aldrich (Cerilliant product)
**Hydrocodone D6**	1007844-38-3	Sigma Aldrich (Cerilliant product)
**Ibuprofen**	15687-27-1	Sigma Aldrich
**Ibuprofen D3**	121662-14-4	Sigma Aldrich (Gillingham, UK)
**Imatinib**	152459-95-5	Sigma Aldrich
**Imazalil sulphate**	58594-72-2	Sigma Aldrich (Gillingham, UK)
**Imidacloprid**	138261-41-3	Sigma Aldrich (PESTANAL)
**Indoprofen**	31842-01-0	Sigma Aldrich (Gillingham, UK)
**Iopromide**	73334-07-3	LGC
**Irbesartan**	138402-11-6	LGC
**Ketamine**	1867-66-9	Sigma Aldrich
**Ketamine D4**	1246815-97-3	LGC standards (Middlesex, UK)
**Ketoprofen**	22071-15-4	Sigma Aldrich
**Lisinopril**	76547-98-3	LGC
**Lomefloxacin**	98079-52-8	Sigma Aldrich
**MDA**	101-77-9	LGC (Cerilliant product)
**MDA D5**	136765-42-9	LGC standards (Middlesex, UK)
**MDMA**	42542-10-9	LGC
**MDMA D5**	136765-43-0	LGC standards (Middlesex, UK)
**MDPV**	687603-66-3	Sigma Aldrich
**Memantine**	19982-08-2	Sigma Aldrich
**Mephedrone**	1189805-46-6	Sigma Aldrich (Cerilliant product)
**Mephedrone D3**	1189972-79-9	LGC standards (Middlesex, UK)
**Metazachlor**	67129-08-2	LGC (Dr Ehrenstorfer)
**Metazachlor D6**	1246816-51-2	Toronto Research Chemicals Inc. (Ontario, Canada)
**Methadone**	76-99-3	Sigma Aldrich (Cerilliant product)
**Methadone D9**	1435933-74-6	LGC standards (Middlesex, UK)
**Methamphetamine**	537-46-2	LGC (Cerilliant product)
**Methamphetamine D5**	60124-88-1	LGC standards (Middlesex, UK)
**Methotrexate**	59-05-2	LGC
**Methylparaben**	99-76-3	Sigma Aldrich
**Methylparaben 13C6**	1581694-95-2	LGC standards (Middlesex, UK)
**Morphine**	57-27-2	Sigma Aldrich (Cerilliant product)
**Morphine D3**	67293-88-3	LGC standards (Middlesex, UK)
**Nalidixic acid**	389-08-2	Sigma Aldrich
**Naproxen**	22204-53-1	Toronto Research Chemicals Inc. (Ontario, Canada)
**N-desmethyltramadol**	75377-45-6	LGC
**N-guanylurea**	207300-86-5	Sigma Aldrich
**Nicotine**	54-11-5	Sigma Aldrich
**Norcodeine**	467-15-2	Sigma Aldrich (Cerilliant product)
**Nordiazepam**	1088-11-5	Sigma Aldrich (Cerilliant product)
**Nordiazepam D5**	65891-80-7	Sigma Aldrich (Cerilliant product)

**Norephedrine**	154-41-6	Sigma Aldrich
**Norfluoxetine**	83891-03-6	LGC (Cerilliant product)
**Normorphine**	466-97-7	Sigma Aldrich (Cerilliant product)
**Noroxycodone**	52446-25-0	LGC
**Nortriptyline**	72-69-5	Sigma Aldrich
**Nortriptyline D3**	203784-52-5	Toronto Research Chemicals Inc. (Ontario, Canada)
**O-6-MAM**	2784-73-8	Sigma Aldrich (Cerilliant product)
**O-desmethyltramadol**	185453-02-5	LGC
**O-desmethylnaproxen**	52079-10-4	Sigma Aldrich (Gillingham, UK)
**Ofloxacin**	82419-36-1	Sigma Aldrich
**Ofloxacin D3**	82419-36-1	Toronto Research Chemicals Inc. (Ontario, Canada)
**Omeprazole**	73590-58-6	Sigma Aldrich (Gillingham, UK)
**Orlistat**	96829-58-2	Sigma Aldrich
**Oxadiazon**	19666-30-9	Sigma Aldrich (PESTANAL)
**Oxycodone**	76-42-6	Sigma Aldrich (Cerilliant product)
**Oxycodone D6**	152477-91-3	Sigma Aldrich (Cerilliant product)
**Oxymorphone**	76-41-5	Sigma Aldrich (Cerilliant product)
**Penicillin G**	61-33-6	Sigma Aldrich
**Penicillin V**	87-08-1	Sigma Aldrich
**Pholcodine**	509-67-1	Sigma Aldrich
**PMA**	3706-26-1	LGC
**Praziquantrel**	55268-74-1	Sigma Aldrich (Gillingham, UK)
**Praziquantrel D11**	1246343-36-1	LGC Standards (Teddington, UK)
**Pregabalin**	148553-50-8	LGC (Cerilliant product)
**Propylparaben**	94-13-3	Sigma Aldrich
**Prulifloxacin**	123447-62-1	Sigma Aldrich
**Quetiapine**	111974-69-7	LGC
**Quetiapine D8**	1185247-12-4	LGC standards (Middlesex, UK)
**Ranitidine**	66357-35-5	Sigma Aldrich
**Risperidone**	106266-06-2	LGC
**Salbutamol**	18559-94-9	Sigma Aldrich (Gillingham, UK)
**Sarafloxacin**	98105-99-8	Sigma Aldrich (VETRANAL)
**Sertraline**	79617-96-2	LGC
**Sertraline D3**	1217741-83-7	Sigma Aldrich (Gillingham, UK)
**Sitagliptin**	486460-32-6	TRC
**Sotalol**	3930-20-9	Sigma Aldrich (Gillingham, UK)
**Sulphadiazine**	68-35-9	Sigma Aldrich (VETRANAL)
**Sulphamethoxazole**	723-46-6	Sigma Aldrich
**Sulphapyridine**	144-83-2	Sigma Aldrich
**Sulphasalazine**	599-79-1	Sigma Aldrich
**Terbutaline**	23031-25-6	Sigma Aldrich (Gillingham, UK)
**Terbuthylazine**	5915-41-3	Sigma Aldrich
**Tetramisole**	5086-74-8	Sigma Aldrich (Gillingham, UK)
**Tetramisole D5**	1173021-85-6	LGC Standards (Teddington, UK)
**Thiamethoxam**	153719-23-4	LGC (Ultra)
**Triallate**	2303-17-5	Sigma Aldrich (PESTANAL)
**Triclosan**	3380-34-5	Sigma Aldrich
**Valsartan**	137862-53-4	Sigma Aldrich
**Vardenafil**	224789-1515-5	Sigma Aldrich (Cerilliant product)
**Venlafaxine**	93413-69-5	Sigma Aldrich
**Zolpidem**	99294-93-6	Sigma Aldrich (Cerilliant product)

Table S2 Method conditions selected for chiral separation

Method	Original column used	Mobile phase B	ABPR (PSI)	Flow rate (mL min^{-1})	Column Temp. (^oC)	Gradient conditions (total run time)	Make-up flow conditions	Ref
A	Amylose 3.0x150 mm, 2.5 µm	1:1 (v/v) IPA:EtOH	1990	2	35	0 min 3 % B, 4 min 30 % B, 6 min 30 % B (9 minutes)	98:2 (% v/v) MeOH/H_2O w/ 0.1 % NH_4OH @ 0.3 mL min^{-1}	(Twohig, O'Leary et al. 2014)
B	AMY-1, CEL-2	1:1:1 (v/v) MeOH:MeCN: IPA	1800	1.5	30	0 min 15 %B, 1 min 15 % B, 5 min 60 % B, 7 min 60 % B (9 minutes)	MeOH w/ 0.1 % formic acid @ 0.3 mL min^{-1}	(Camacho-Munoz, Kasprzyk-Hordern et al. 2016)
C		1:1 (v/v) EtOH:MeCN with 0.2 % (v/v) TFA	1800	1.5	30	0 min 5 % B, 3.5 min 5 % B, 10 min 60 % B, 13.5 min 60 % B (16 minutes)	MeOH w/ 0.2 % NH_4OH @ 0.3 mL min^{-1}	
D	CHIRAL PAK IB-3, 2.1x100 mm	20 mM NH_4OAc in MeOH	1500	2	35	0 min 7 % B, 1 min 7 % B, 7 min 25 % B, 9 min 25 % B (10 minutes)	Not given, conditions for method A used	(Waters 2012)

Table S3 Matrix of method conditions used to analyse analytes

SFC method / Column	AMY-1	CEL-1	CEL-2	Mobile phase conditions
**A**	A1	A2	A3	B: IPA:EtOH – 9 minute gradient
**B**	B1	B2	B3	B: MeOH:MeCN:IPA – 9 minute gradient
**C**	C1	C2	C3	B: EtOH:MeCN with 0.2 % TFA – 16 minute gradient
**D**	D1	D2	D3	B: MeOH with 20 mM NH_4OAC – 10 minute gradient

Table S4 Chiral separation using twelve different methods

Method / column	AMY-1	CEl-1	CEL-2
**A**	A1: Temazepam, tramadol (partial)	A2: Bisprolol, Metoprolol, propranolol, ketamine, Mepehdrone (partial), Methamphetamine (partial), MDMA (partial)	A:3 Temazepam
**B**	B1: Bisoprolol, temazepam, metoprolol, propranolol, ketamine, desmethylvenlafaxine, PMA (partial), Mephedrone (partial)	B:2 Bisprolol, metoprolol, atenolol, propranolol, temazepam (partially), PMA	B3: Bisoprolol, temazepam, atenolol, ketamine, propranolol (partial)
**C**	C1: Metoprolol, propranolol, EDDP	C2: Bisoprolol, temazepam, metoprolol (partially), atenolol, propranolol, ketamine, mephedrone (partially), fluoxetine	C3: Temazepam, atenolol
**D**	D1: Ketamine, methamphetamine (partial), Fluoxetine, betablockers separated but carry-over into the next injection	D2: Mephedrone and methamphetamine, MDMA (partial), PMA (partial)	D3: No compounds separated

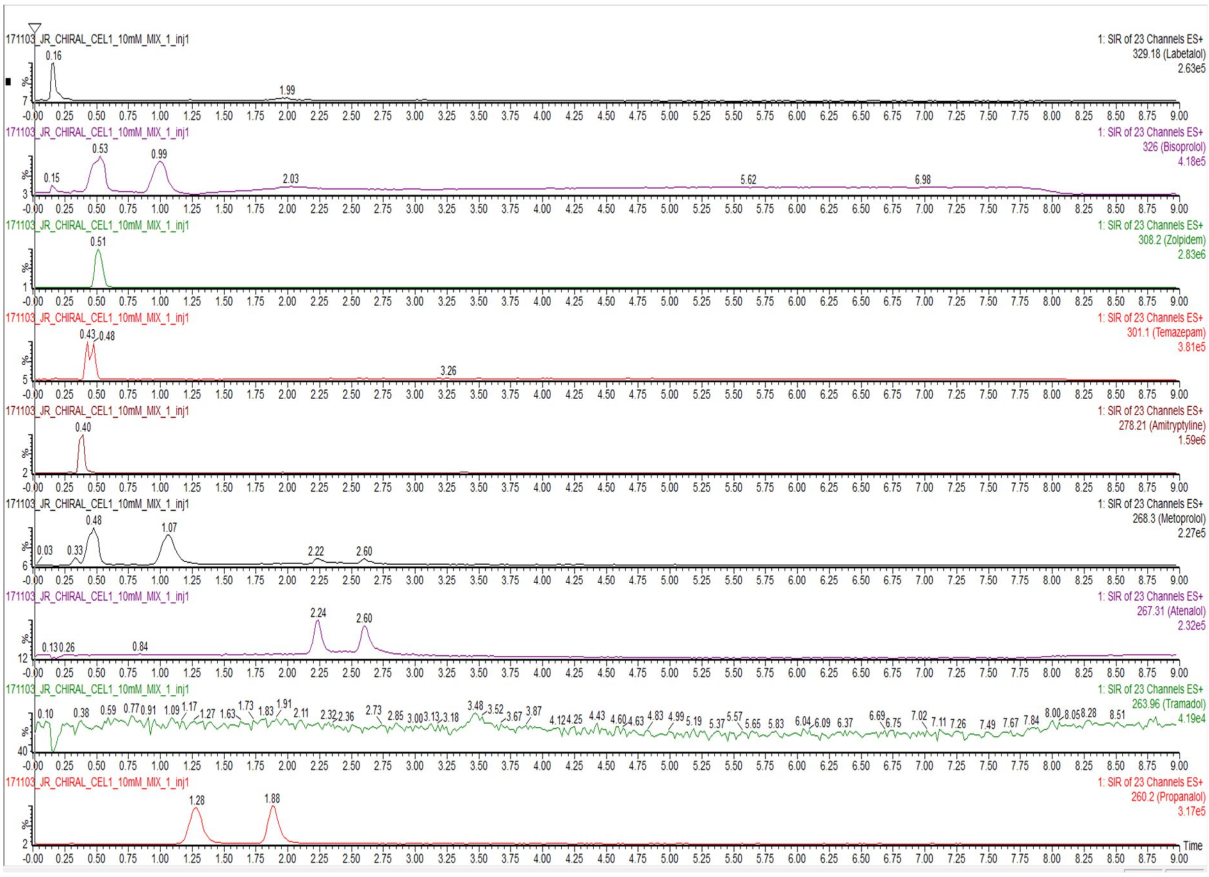

Fig. S1 Enantioselective separation of bisoprolol, temazepam (partial), metoprolol, atenolol and propranolol in method B2. N.B. Labetolol elution occurred during column void volume

Table S5 MRM conditions of analytes and isotopically labelled internal standards studied using the selected SFC-TQD method

Analyte	MRM 1 transition	CV (V) / CE (V)	MRM 2 transition	CV (V) / CE (V)
**1,7 dimethylxantine**	181 > 124	54 / 21	-	-
**1S,2R-(+)-ephedrine D3**	169 > 151	23 / 18	-	-
**2-Hydroxyibuprofen**	221 > 177	30 /6	221 > 159	30 / 12
**2-Phenylproponic Acid**	149 > 105	20 / 5	-	-
**8-isoF2B**	353 > 309	53 / 18	353 > 247	53 / 22
**Acetaminophen**	152 > 110	26 / 16	152 > 93	26 / 24
**Acetaminophen D4**	156 > 114	26 / 16	-	-
**AEME**	182 > 118	39 / 23	182 > 122	39 /21
**Aminorex**	163 > 120	20 / 15	163 > 103	20 / 20
**Amoxicillin**	366 > 208	22 / 13	366 > 114	22 / 23
**Amphetamine**	136 > 119	18 / 8	136 > 91	18 / 16
**Amphetamine D5**	141 > 93	20 / 14	-	-
**Ampicillin**	350 > 160	44 / 10	350 > 107	44 / 28
**Azathioprine**	278 > 142	28 / 20	278 > 85	28 / 13
**Azithromycin**	750 > 116	60 / 54	750 > 83	60 / 60
**Benzophenone-1**	213 > 135	36 / 20	213 > 91	34 / 25
**Benzophenone-2**	245 > 109	32 / 20	245 > 135	32 / 13
**Benzophenone-3**	229 > 151	35 / 18	229 > 105	35 / 20
**Benzophenone-4**	307 > 227	44 / 24	307 > 211	42 / 35
**Benzoylecgonine**	290 > 168	38 / 19	290 > 105	38 / 30
**Benzoylecgonine D8**	298 > 171	38 / 19	-	-
**Benzylpiperizine**	177 > 91	35 / 20	177 > 85	35 / 15
**Bezafibrate**	360 > 274	30 / 19	360 > 154	30 / 28
**Bicalutamide**	431 > 217	40 / 15	431 > 187	40 / 13
**Buprenorphine**	468 > 396	66 / 41	468 > 414	66 / 35
**Caffeine**	195 > 138	38 / 15	195 > 110	38 / 23
**Candesartan Cilexetil**	611 > 567	44 / 7	611 > 467	44 / 7
**Capecitabine**	360 > 244	25 / 11	360 > 174	25 / 23
**Carbamazepine**	237 > 194	40 / 20	237 > 179	40 / 38
**Carbamazepine-10,11-epoxide**	253 > 210	39 / 12	253 > 180	39 / 25
**Carbamazepine 13C6**	243 > 200	40 / 20	-	-
**Carboxyibuprofen**	235 > 191	25 / 8	235 > 73	25 / 15
**Carprofen**	272 > 228	39 / 17	272 > 226	39 / 39
**Cetirizine**	389 > 201	32 / 21	389 > 166	32 / 40
**Chloramphenicol**	321 > 152	27 / 15	321 > 194	35 / 12
**Chlorpyrifos**	350 > 198	34 / 16	350 > 125	34 / 19
**Cimetidine**	253 > 159	22 / 16	253 > 211	22 / 10
**Citalopram**	325 > 262	46 / 18	325 > 110	46 / 26
**Citalopram D6**	331 > 109	46 / 28	-	-
**Clarithromycin**	749 > 590	40 / 20	749 > 158	40 / 31
**Clothiniadin**	250 > 132	28 / 15	250 > 113	28 / 25
**Cocaethylene**	318 > 196	38 / 20	318 > 82	38 / 30
**Cocaethylene D3**	321 > 199	40 / 22	-	-
**Cocaine**	304 > 182	40 / 20	304 > 82	40 / 31
**Cocaine D3**	307 > 185	40 / 20	-	-
**Codeine**	300 > 215	49 / 25	300 > 152	49 / 57
**Codeine D6**	306 > 218	52 / 28	-	-
**Cotinine**	177 > 80	34 / 21	177 > 98	34 / 22
**Cotinine D3**	180 > 80	44 / 24	-	-
**Creatinine**	114 > 44	30 / 15	114 > 86	31 / 11
**Cytarabine**	244 > 112	20 / 14	244 > 133	20 / 15
**Danofloxacin**	358 > 340	65 / 20	358 > 255	38 / 38
**Desmethylcitalopram**	311 > 109	46 / 27	311 > 262	46 / 18
**Desmethylvenlafaxine**	264 > 107	25 / 24	264 > 246	25 / 20
**Desvenlafaxine**	264 > 107	25 / 24	264 > 246	25/ 20
**DHMA**	182 > 123	6 / 18	182 > 151	6 / 12
**Diazepam**	285 > 154	56 / 29	285 > 222	56 / 27
**Diazepam D5**	290 > 198	56 / 34	-	-
**Diazinon**	305 > 169	36 / 22	305 > 153	36 / 22
**Dichlofluanid**	335 > 123	29 / 31	335 > 271	29 / 6
**Diclofenac**	294 > 250	22 / 13	-	-
**Dihydrocodeine**	302 > 199	53 / 33	302 > 128	53 / 60
**Dihydroketoprofen**	255 > 211	30 / 8	-	-
**Dihydromorphine**	288 > 185	28 / 42	288 > 213	28 / 32
**Diltiazem**	415 > 178	40 / 25	415 > 310	40 / 25
**Donepezil**	380 > 288	56 / 24	380 > 262	56 / 23
**Duloxetine**	298 > 154	16 / 5	280 > 188	16 / 5
**E1 & E2-10,11-dihydro-10-hydroxycarbamazepine**	255 > 194	20 / 20	255 > 179	20 / 40

**E1 & E2-Alprenolol**	250 > 116	44 / 16	250 > 98	44 / 18
**E1 & E2-Atenolol**	267 > 145	38 / 30	267 > 190	38 / 16
**E1 & E2-Atenolol D7**	274 > 145	44 / 30	-	-
**E1 & E2-Bisoprolol**	326 > 116	45 / 18	326 > 204	45 / 19
**E1 & E2-Metoprolol**	268 > 116	42 / 20	268 > 121	42 / 22
**E1 & E2-Mirtazapine**	266 > 195	44 / 18	266 > 72	44 / 26
**E1 & E2-Mirtazapine D3**	269 > 195	35 / 25	-	-
**E1 & E2-Propanolol**	260 > 116	42 / 16	260 > 183	42 / 18
**E1 & E2-Tramadol**	264 > 58	28 / 45	264 > 121	28 / 46
**E1& E2-Metoprolol D7**	275 > 123	44 / 20	-	-
**E1& E2-Oxazepam**	287 > 241	38 / 22	287 > 269	38 /14
**E1& E2-Oxazepam D5**	292 > 274	38 / 15	-	-
**E1& E2-Propanolol D7**	267 > 189	40 / 18	-	-
**Ecgonine methylester D3**	203 > 85	44 / 22	-	-
**EE2**	295 > 159	60 / 40	295 > 145	60 / 40
**Ephedrine**	166 > 148	23 / 12	166 > 133	23 / 21
**Erythromycin**	735 > 159	12 / 32	735 > 576	12 / 20
**Ethylparaben**	165 > 92	26 / 20	164 > 137	20 / 14
**Fexofenadine**	500 > 456	33 / 14	500 > 378	33 / 19
**Flufenacet**	364 > 194	24 / 10	364 > 152	24 / 18
**Flumequine**	262 > 202	28 / 34	262 > 245	28 / 26
**Fluoxetine**	310 > 44	34 / 10	310 > 148	34 / 10
**Fluoxetine D5**	315 > 153	26 / 8	-	-
**Furosemide**	331 > 313	31 / 8	331 > 239	31 / 10
**Gabapentin**	172 > 154	30 / 12	172 > 95	30 / 22
**Gabapentin D4**	176 > 158	33 / 16	-	-
**Gemfibrozil**	251 > 205	21 / 9	251 > 123	21 / 14
**Gliclazide**	324 > 127	41 / 20	324 > 110	41 / 20
**Griseofulvin**	353 > 69	45 / 25	353 > 165	45 / 23
**Heroin**	370 > 165	51 / 50	370 > 268	51 / 29
**Heroin D9**	379 > 166	51 / 50	-	-
**HMA**	182 > 123	6 / 18	182 > 165	6 / 14
**HMMA**	196 > 165	16 / 12	196 > 133	16 / 22
**HNE-MA**	318 > 171	32 / 22	318 > 162	32 / 14
**Hydrocodone**	300 > 199	24 / 34	300 > 171	24 / 46
**Hydrocodone D6**	306 > 202	64 / 32	-	-
**Ibuprofen**	205 > 161	19 / 8	-	-
**Ibuprofen D3**	208 > 164	20 / 6	-	-
**Imatinib**	494 > 394	57 / 27	494 > 378	57 / 48
**Imazalil sulphate**	297 > 159	40 / 20	297 > 201	40 / 18
**Imidacloprid**	256 > 209	34 / 15	256 > 175	34 / 19
**Indoprofen**	282 > 236	45 / 20	282 > 77	45 / 15
**Iopromide**	792 > 573	46 / 25	792 > 559	46 / 32
**Irbesartan**	427 > 193	50 / 28	427 > 121	50 / 65
**Ketamine**	238 > 125	31 / 27	238 > 220	31 / 15
**Ketamine D4**	242 > 129	31 / 27	-	-
**Ketoprofen**	253 > 209	15 / 7	253 > 212	15 / 7
**Lisinopril**	406 > 84	38 / 27	406 > 246	38 / 22
**Lomefloxacin**	352 > 265	22 / 24	352 > 308	22 / 22
**MDA**	180 > 163	21 / 11	180 > 105	21 / 22
**MDA D5**	185 > 168	21 / 11	-	-
**MDMA**	194 > 163	24 / 13	194 > 105	24 / 24
**MDMA D5**	199 > 165	26 /13	-	-
**MDPV**	276 > 126	40 / 28	276 > 135	40 / 25
**Memantine**	180 > 107	36 / 24	180 > 121	36 / 24
**Mephedrone**	178 > 145	10 / 22	178 > 160	10 / 12
**Mephedrone D3**	181 > 148	30 / 22	-	-
**Metazachlor**	278 > 210	21 / 21	278 > 134	21 / 10
**Metazachlor D6**	284 > 216	21 / 10	-	-
**Methadone**	310 > 265	31 / 15	310 > 105	31 /28
**Methadone D7**	319 > 268	31 / 15	-	-
**Methamphetamine**	150 > 91	24 / 19	150 > 119	24 / 10
**Methamphetamine D5**	155 > 92	28 / 18	-	-
**Methotrexate**	455 > 175	40 / 35	455 > 308	40 / 20
**Methylparaben**	151 > 92	34 / 20	151 > 136	20 / 14
**Methylparaben 13C6**	157 > 98	30 / 20	-	-
**Morphine**	286 > 165	53 / 38	286 > 152	53 / 56
**Morphine D3**	289 > 152	53 / 56	-	-
**Nalidixic acid**	233 > 187	30 / 28	233 > 131	30 / 36
**Naproxen**	229 > 169	20 / 8	229 > 185	20 / 8
**N-desmethyl tramadol**	250 > 44	25 / 12	250 > 232	25 / 8
**N-Guanylurea**	103 > 60	24 / 10	103 > 86	24 / 8
**Nicotine**	163 > 130	37 / 20	163 > 117	37 / 24
**Norcodeine**	286 > 165	46 / 40	286 > 268	46 / 20

**Nordiazepam**	271 > 140	51 / 29	271 > 165	51 / 29
**Nordiazepam D5**	276 > 140	48 / 36	-	-
**Norephedrine**	152 > 134	23 / 10	152 > 117	23 / 16
**Norfluoxetine**	296 > 134	18 / 6	-	-
**Normorphine**	272 > 165	45 / 43	272 > 152	45 / 49
**Noroxycodone**	302 > 227	22 / 36	302 > 187	22 /38
**Nortriptyline**	264 > 91	33 / 23	264 > 233	33 / 16
**Nortriptyline D3**	267 > 191	40 / 20	267 > 233	40 / 8
**O-6-MAM**	328 > 165	52 / 39	328 > 211	52 / 26
**O-desmethyl tramadol**	250 > 58	30 > 18	250 > 232	30 / 10
**O-Desmethylnaproxen**	215 > 170	20 / 10	-	-
**Ofloxacin**	362 > 261	43 / 28	362 > 318	43 / 19
**Ofloxacin D3**	365 > 261	47 / 28	365 > 322	47 / 23
**Omeprazole**	346 > 198	20 / 10	-	-
**Orlistat**	496 > 319	40 / 13	496 > 160	40 / 12
**Orlistat**	496 > 319	40 / 13	496 > 160	40 / 12
**Oxadiazon**	345 > 303	43 / 14	344.9 > 302.8	43 / 20
**Oxycodone**	316 > 241	36 / 29	316 > 256	36 / 26
**Oxycodone D6**	322 > 247	36 / 29	-	-
**Oxymorphone**	302 > 284	40 / 19	302 > 227	40 / 28
**Penicillin G**	335 > 176	48 / 20	335 > 160	48 / 20
**Penicillin V**	316 > 114	54 / 40	316 > 160	54 / 40
**Pholcodine**	399 > 381	55 / 25	399 > 100	55 / 35
**PMA**	166 > 121	20 / 20	166 > 149	20 / 20
**Praziquantrel**	313 > 203	40 / 15	313 > 83	40 / 35
**Praziquantrel D11**	325 > 204	40 / 20	-	-
**Pregabalin**	160 > 142	32 / 11	160 > 125	32 / 14
**Propylparaben**	179 > 92	34 / 25	179 > 136	20 / 16
**Prulifloxacin**	462 > 444	42 / 22	462 > 360	42 / 32
**Quetiapine**	384 > 253	50 / 21	384 > 221	50 / 40
**Quetiapine D8**	392 > 258	50 / 23	-	-
**Ranitidine**	316 > 176	26 / 17	316 > 124	26 / 14
**Risperidone**	411 > 191	49 / 30	411 > 110	49 / 51
**Salbutamol**	240 > 148	30 / 18	240 > 166	30 / 14
**Sarafloxacin**	386 > 368	49 / 23	386 > 299	49 / 28
**Sertraline**	306 > 159	23 / 27	306 > 275	23 / 10
**Sertraline D3**	309 > 159	23 / 27	-	-
**Sitagliptin**	408 > 235	46 / 19	408 > 193	46 / 26
**Sotalol**	273 > 133	30 / 28	273 > 213	30 / 16
**Sulphadiazine**	251 > 108	30 / 26	251 > 158	30 / 15
**Sulphamethoxazole**	254 > 92	36 / 30	254 > 156	36 / 20
**Sulphapyridine**	250 > 156	42 / 16	250 > 92	42 / 30
**Sulphasalazine**	397 > 197	45 / 25	397 > 240	45 / 25
**Terbutaline**	226 > 105	66 / 22	-	-
**Terbuthylazine**	230 > 174	35 / 17	230 > 132	35 / 24
**Tetramisole**	205 > 91	45 / 35	205 > 123	45 / 30
**Tetramisole D5**	211 > 183	30 / 15	-	-
**Thiamethoxam**	292 > 211	44 / 12	292 > 132	44 / 22
**Triallate**	306 > 145	34 / 26	306 > 128	34 / 13
**Triclosan**	289 > 35	18 / 10	289 > 37	18 / 10
**Valsartan**	434 > 350	35 / 20	434 > 179	35 / 25
**Vardenafil**	489 > 151	74 / 68	489 > 312	74 / 48
**Venlafaxine**	278 > 58	27 / 40	278 > 260	27 / 12
**Zolpidem**	308 > 221	8 / 44	-	-

Table S6 Average analyte retention times in each of the three methods (n=42)

PHARMA method analyte	t_R (min)	SD	DAC method analyte	t_R (min)	SD	NEG method analyte	t_R (min)	SD
1,7-DMX	0.8	0.01	AEME<br>Ecgoninemethylester D3	0.3	0.0	2-Hydroxy-ibuprofen	1.1	0.04
Acetaminophen<br>Acetaminophen D4	0.8	0.02	Aminorex	1.3	0.03	2-Phenylproponic acid	N/A	0.03
Azathioprine	2.5	0.02	Amoxicillin	3.4	0.05	8-IsoF2B	2.7	0.02
Benzophenone-3	0.4	0.01	Amphetamine<br>Amphetamine D5	0.8	0.00	Benzophenone-1	0.6	0.01
Bicalutamide	0.7	0.02	Ampicillin	2.9	0.05	Benzophenone-2	N/A	N/A
Buprenorphine	0.9	0.01	Azithromycin	1.2	0.05	Benzophenone-4	4.5	0.01
Caffeine	0.6	0.00	Benzoylecgonine<br>Benzoylecgonine D8	2.4	0.01	Bezafibrate	3.8	0.01
Candesartan Cilexetil	3.4	0.01	Benzylpiperizine	1.0	0.01	Carboxyibuprofen	4.0	0.04
Capecitabine	1.4	0.02	Carbamazepine<br>Carbamazepine $^{13}C_6$	1.6	0.00	Carprofen	3.1	0.02
Chlorpyrifos	0.4	0.01	Carbamazepine-10,11-epoxide	2.1	0.01	Chloramphenicol	1.0	0.01
Cimetidine	2.2	0.03	Cetirizine	3.2	0.05	Diclofenac	2.4	0.01
Citalopram<br>Citalopram D6	0.7	0.01	Clarithromycin	1.2	0.05	Dihydro-ketoprofen	2.5	0.02
Clothiniadin	1.5	0.02	Cocaethylene<br>Cocaethylene D3	0.4	0.00	EE2	N/A	N/A
Codeine<br>Codeine D6	1.2	0.01	Cocaine<br>Cocaine D3	0.4	0.00	Ethylparaben	0.4	0.01
Cotinine<br>Cotinine D3	0.5	0.00	Danofloxacin	3.0	0.05	Fexofenadine	4.2	0.02
Creatinine	2.0	0.04	DHMA	0.4	0.01	HNE-MA	3.9	0.02
Cytarabine	3.6	0.02	E1-10,11-dihydro-<br>10-hydroxy-carbamazepine	1.4	0.01	Ibuprofen<br>Ibuprofen D_3	0.5	0.02
Desmethyl-citalopram	1.2	0.01	E1-Alprenolol	0.8	0.01	Irebsartan	3.4	0.00
Desmethyl-venlafaxine	1.1	0.01	E1-Atenolol<br>E1-Atenolol D7	3.1	0.01	Ketoprofen	1.2	0.02
Desvenlafaxine	1.1	0.01	E1-Bisoprolol	1.1	0.00	Methylparaben<br>Methylparaben $^{13}C_6$	0.4	0.01
Diazepam<br>Diazepam D5	0.9	0.01	E1-Metoprolol<br>E1-Metoprolol D7	1.0	0.02	Naproxen	N/A	N/A
Diazinon	0.3	0.00	E1-Propanolol<br>E1-Propanolol D7	2.3	0.01	O-desmethyl-naproxen	2.9	0.01
Dichlofluanid	0.4	0.01	E1-Tramadol	0.6	0.00	Propylparaben	0.4	0.01
Dihydrocodeine	1.2	0.01	E2-10,11-dihydro-<br>10-hydroxy-carbamazepine	1.8	0.01	Sulphasalazine	5.5	0.02
Dihydromorphine	2.6	0.02	E2-Alprenolol	1.1	0.01	Triclosan	0.6	0.01
Diltiazem	0.6	0.00	E2-Atenolol<br>E2-Atenolol D7	3.4	0.00	Valsartan	7.1	0.04
Duloxetine	1.8	0.01	E2-Bisoprolol	2.0	0.01			
E1-Mirtazapine<br>E1-Mirtazapine D3	0.7	0.01	E2-Metoprolol<br>E2-Metoprolol D7	2.1	0.01			
E1-Oxazepam<br>E1-Oxazepam D5	2.0	0.01	E2-Propanolol<br>E2-Propanolol D7	2.8	0.01			
E2-Mirtazapine<br>E2-Mirtazapine D3	0.8	0.01	E2-Tramadol	1.1	0.00			
E2-Oxazepam<br>E2-Oxazepam D5	2.4	0.01	Ephedrine	1.0	0.02			
Flufenacet	0.4	0.01	Erythromycin	0.9	0.05			
Fluoxetine<br>Fluoxetine D5	0.6	0.01	Flumequine	1.5	0.05			
Furosemide	0.5	0.01	Griseofulvin	0.8	0.00			
Gabapentin<br>Gabapentin D4	3.6	0.02	Heroin<br>Heroin D9	0.5	0.01			
Gemfibrozil	0.4	0.02	HMA	2.1	0.01			
Gliclazide	1.0	0.02	HMMA	1.7	0.01			
Hydrocodone<br>Hydrocodone D6	1.1	0.02	Imazalil sulphate	0.6	0.02			
Imatinib	3.8	0.01	Indoprofen	2.7	0.01			
Imidacloprid	1.5	0.01	Ketamine<br>Ketamine D4	0.5	0.00			
Iopromide	3.9	0.03	Lomefloxacin	3.2	0.05			
Memantine	1.0	0.01	MDA<br>MDA D5	1.0	0.03			
Metazachlor	0.4	0.01	MDMA<br>MDMA D5	0.8	0.01			
Morphine<br>Morphine D3	2.2	0.02	MDPV	0.4	0.01			
N-guanylurea	2.3	0.02	Mephedrone<br>Mephedrone D3	0.4	0.01			
Nicotine	0.4	0.01	Methadone	0.6	0.00			

Norcodeine	2.8	0.02	Methadone D7<br>Methamphetamine<br>Methamphetamine D5	0.7	0.00
Nordiazepam<br>Nordiazepam D5	1.5	0.01	Nalidixic acid	1.0	0.05
Norfluoxetine	1.0	0.01	N-desmethyl-tramadol	1.2	0.03
Normorphine	3.5	0.02	Norephedrine	1.4	0.01
Noroxycodone	2.7	0.03	O-6-MAM	1.0	0.02
Nortriptyline<br>Nortryptyline D3	1.3	0.02	O-desmethyl-tramadol	1.1	0.01<br>0.01
Omeprazole	1.2	0.01	Ofloxacin<br>Ofloxacin D3	2.7	0.01
Orlistat	0.4	0.01	Orlistat	0.5	0.02
Oxadiazon	0.3	0.01	Penicillin G	3.4	0.05
Oxycodone<br>Oxycodone D6	0.7	0.01	Penicillin V	3.0	0.05
Oxymorphone	2.7	0.02	PMA	0.9	0.02
Pholcodine	2.2	0.01	Praziquantrel<br>Praziquantrel D11	1.0	0.01
Pregabalin	3.6	0.02	Prulifloxacin	3.4	0.05
Quetiapine<br>Quetiapine D8	1.3	0.01	Sarafloxacin	3.2	0.05
Ranitidine	2.6	0.01	Sulphadiazine	2.4	0.00
Risperidone	1.9	0.01	Sulphamethoxazole	1.8	0.01
Salbutamol	3.0	0.02	Sulphapyridine	2.3	0.00
Sertraline<br>Sertraline D3	1.1	0.01	Tetramisole<br>Tetramisole D5	0.8	0.00
Sitagliptin	1.4	0.03	Tylosin	N/A	N/A
Sotalol	2.5	0.01			
Terbutaline	0.6	0.00			
Terbuthylazine	0.4	0.00			
Thiamethoxam	1.4	0.02			
Triallate	0.4	0.02			
Vardenafil	3.2	0.02			
Venlafaxine	0.5	0.00			
Zolpidem	1.0	0.01			

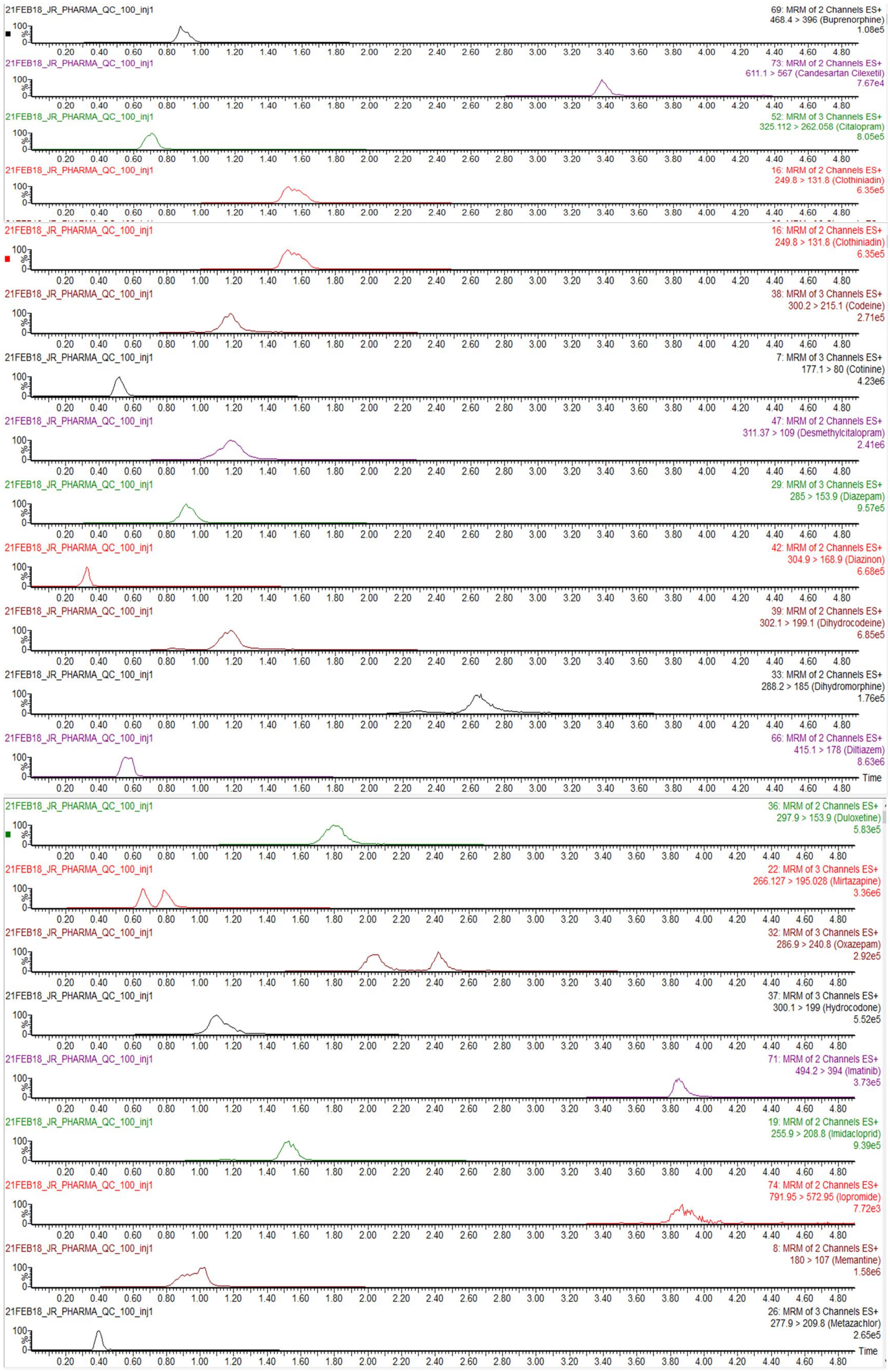
21FEB18_JR_PHARMA_QC_100_inj1
69: MRM of 2 Channels ES+
468.4 > 396 (Buprenorphine)
1.08e5
73: MRM of 2 Channels ES+
611.1 > 567 (Candesartan Cilexetil)
7.67e4
52: MRM of 3 Channels ES+
325.112 > 262.058 (Citalopram)
8.05e5
16: MRM of 2 Channels ES+
249.8 > 131.8 (Clothiniadin)
6.35e5
38: MRM of 3 Channels ES+
300.2 > 215.1 (Codeine)
2.71e5
7: MRM of 3 Channels ES+
177.1 > 80 (Cotinine)
4.23e6
47: MRM of 2 Channels ES+
311.37 > 109 (Desmethylcitalopram)
2.41e6
29: MRM of 3 Channels ES+
285 > 153.9 (Diazepam)
9.57e5
42: MRM of 3 Channels ES+
304.9 > 168.9 (Diazinon)
6.68e5
39: MRM of 3 Channels ES+
302.1 > 199.1 (Dihydrocodeine)
6.85e5
33: MRM of 2 Channels ES+
288.2 > 185 (Dihydromorphine)
1.76e5
66: MRM of 2 Channels ES+
415.1 > 178 (Diltiazem)
8.63e6
36: MRM of 2 Channels ES+
297.9 > 153.9 (Duloxetine)
5.83e5
22: MRM of 3 Channels ES+
266.127 > 195.028 (Mirtazapine)
3.36e6
32: MRM of 3 Channels ES+
286.9 > 240.8 (Oxazepam)
2.92e5
37: MRM of 3 Channels ES+
300.1 > 199 (Hydrocodone)
5.52e5
71: MRM of 2 Channels ES+
494.2 > 394 (Imatinib)
3.73e5
19: MRM of 2 Channels ES+
255.9 > 208.8 (Imidacloprid)
9.39e5
74: MRM of 2 Channels ES+
791.95 > 572.95 (Iopromide)
7.72e3
8: MRM of 2 Channels ES+
180 > 107 (Memantine)
1.58e6
26: MRM of 3 Channels ES+
277.9 > 209.8 (Metazachlor)
2.65e5
Time

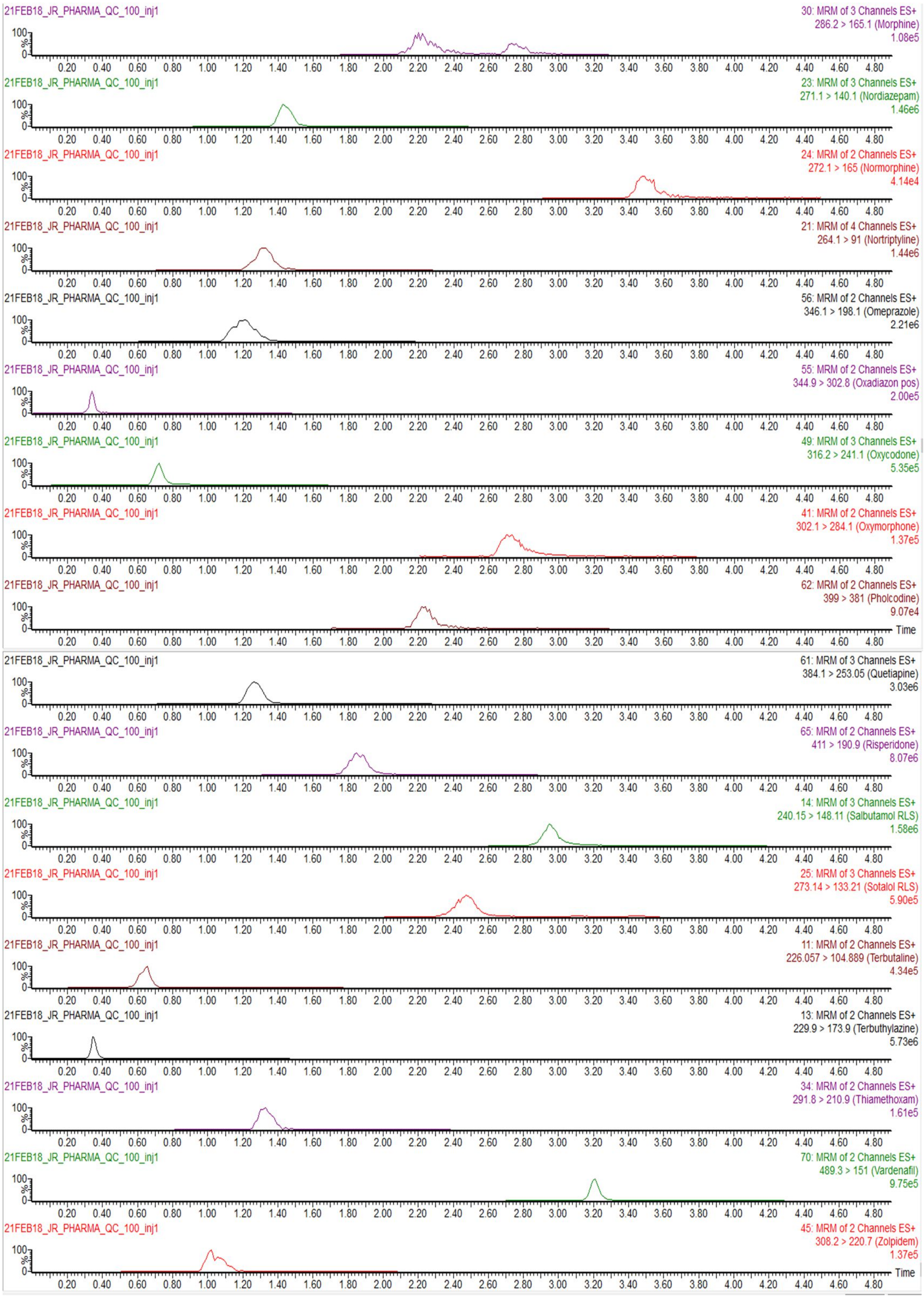

Fig. S2 Extracted mass chromatograms for analytes in the PHARMA method using their MRM 1 transitions

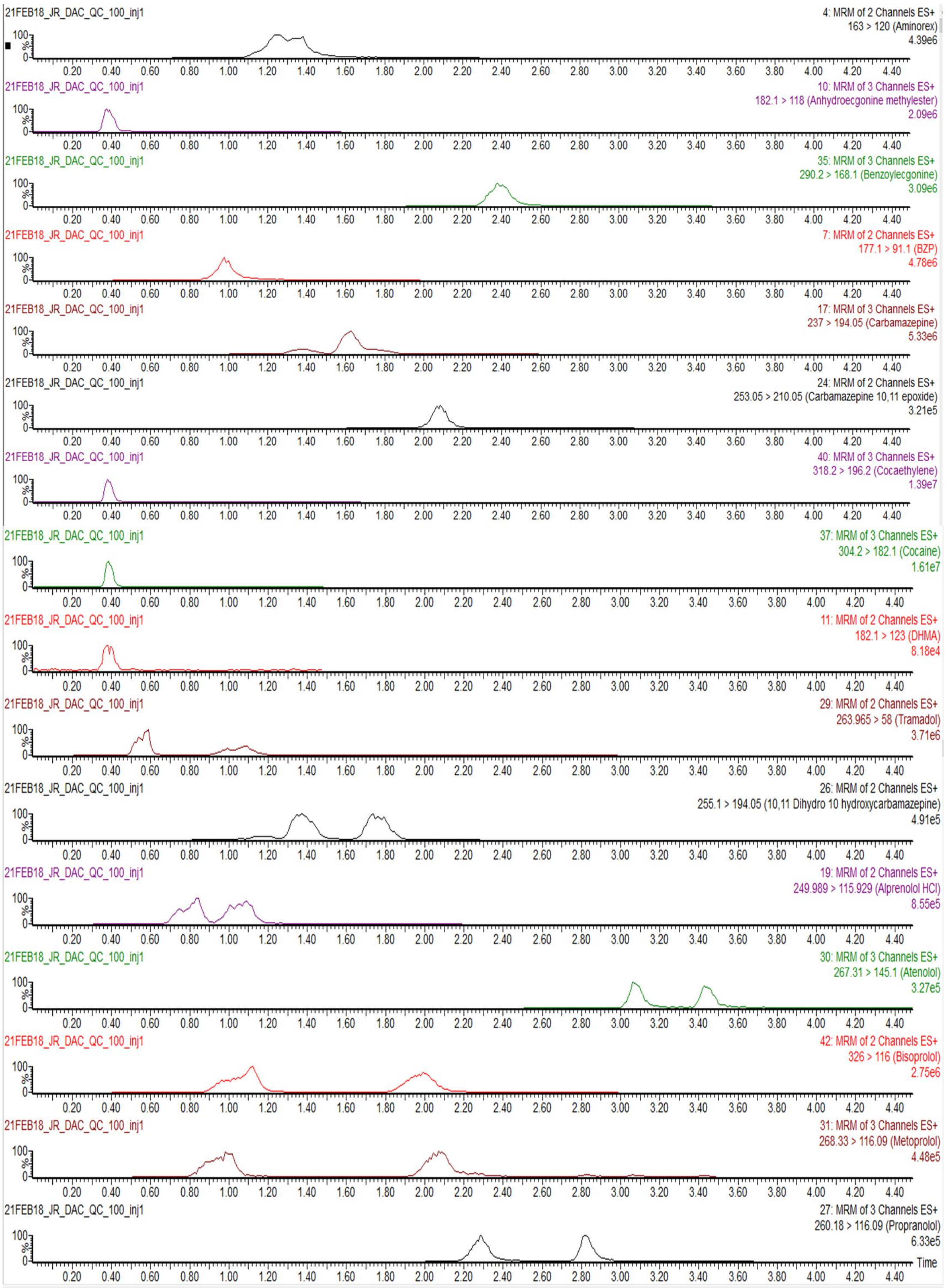
21FEB18_JR_DAC_QC_100_inj1
4: MRM of 2 Channels ES+
163 > 120 (Aminorex)
4.39e6
21FEB18_JR_DAC_QC_100_inj1
10: MRM of 3 Channels ES+
182.1 > 118 (Anhydroecgonine methylester)
2.09e6
21FEB18_JR_DAC_QC_100_inj1
35: MRM of 3 Channels ES+
290.2 > 168.1 (Benzoylecgonine)
3.09e6
21FEB18_JR_DAC_QC_100_inj1
7: MRM of 2 Channels ES+
177.1 > 91.1 (BZP)
4.78e6
21FEB18_JR_DAC_QC_100_inj1
17: MRM of 3 Channels ES+
237 > 194.05 (Carbamazepine)
5.33e6
21FEB18_JR_DAC_QC_100_inj1
24: MRM of 2 Channels ES+
253.05 > 210.05 (Carbamazepine 10,11 epoxide)
3.21e5
21FEB18_JR_DAC_QC_100_inj1
40: MRM of 3 Channels ES+
318.2 > 196.2 (Cocaethylene)
1.39e7
21FEB18_JR_DAC_QC_100_inj1
37: MRM of 3 Channels ES+
304.2 > 182.1 (Cocaine)
1.61e7
21FEB18_JR_DAC_QC_100_inj1
11: MRM of 2 Channels ES+
182.1 > 123 (DHMA)
8.18e4
21FEB18_JR_DAC_QC_100_inj1
29: MRM of 3 Channels ES+
263.965 > 58 (Tramadol)
3.71e6
21FEB18_JR_DAC_QC_100_inj1
26: MRM of 2 Channels ES+
255.1 > 194.05 (10,11 Dihydro 10 hydroxycarbamazepine)
4.91e5
21FEB18_JR_DAC_QC_100_inj1
19: MRM of 2 Channels ES+
249.989 > 115.929 (Alprenolol HCl)
8.55e5
21FEB18_JR_DAC_QC_100_inj1
30: MRM of 3 Channels ES+
267.31 > 145.1 (Atenolol)
3.27e5
21FEB18_JR_DAC_QC_100_inj1
42: MRM of 2 Channels ES+
326 > 116 (Bisoprolol)
2.75e6
21FEB18_JR_DAC_QC_100_inj1
31: MRM of 3 Channels ES+
268.33 > 116.09 (Metoprolol)
4.48e5
21FEB18_JR_DAC_QC_100_inj1
27: MRM of 3 Channels ES+
260.18 > 116.09 (Propranolol)
6.33e5
100
%
0
0.20 0.40 0.60 0.80 1.00 1.20 1.40 1.60 1.80 2.00 2.20 2.40 2.60 2.80 3.00 3.20 3.40 3.60 3.80 4.00 4.20 4.40
Time

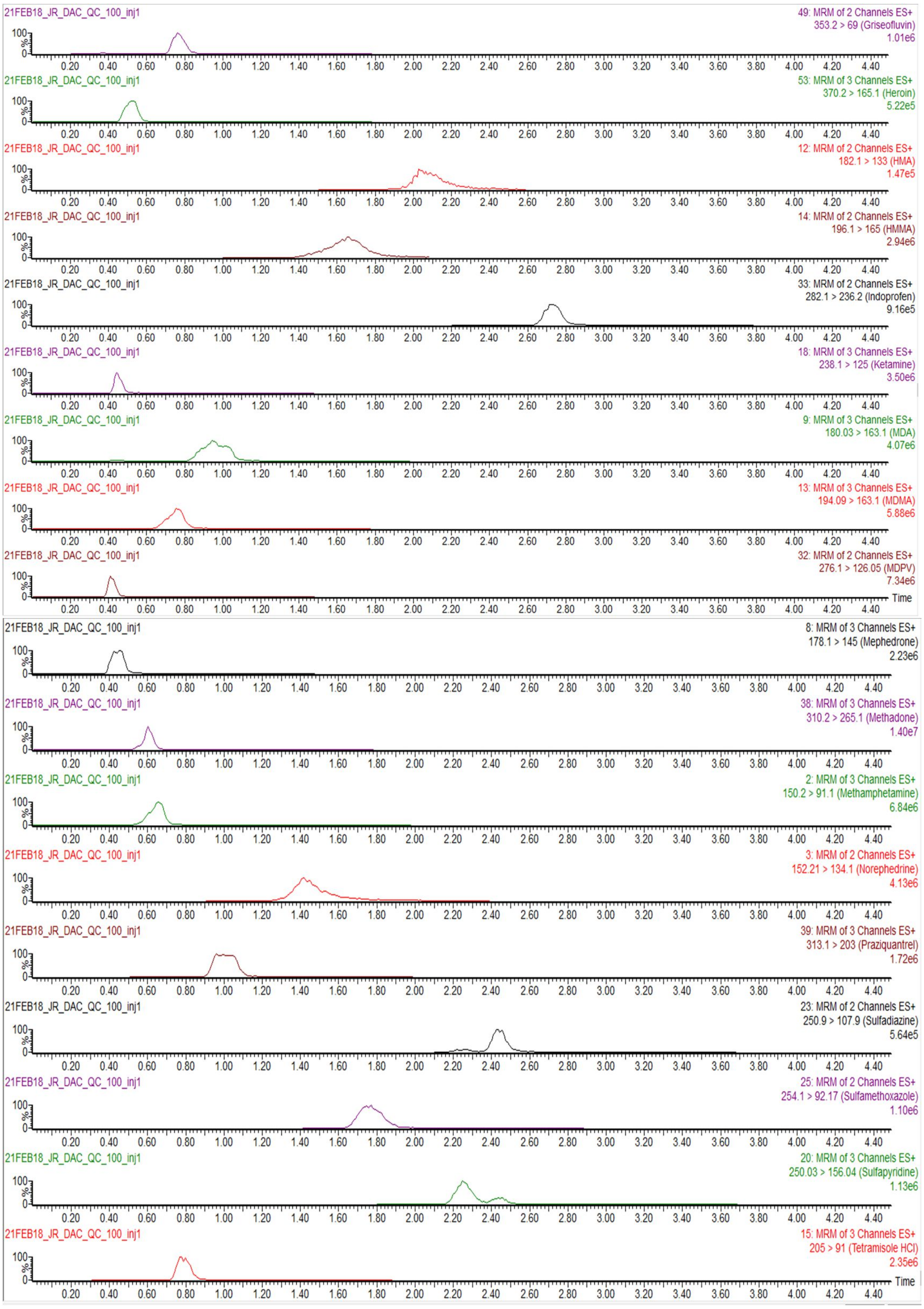

Fig. S3 Extracted mass chromatograms for analytes in the DAC method using their MRM 1 transitions

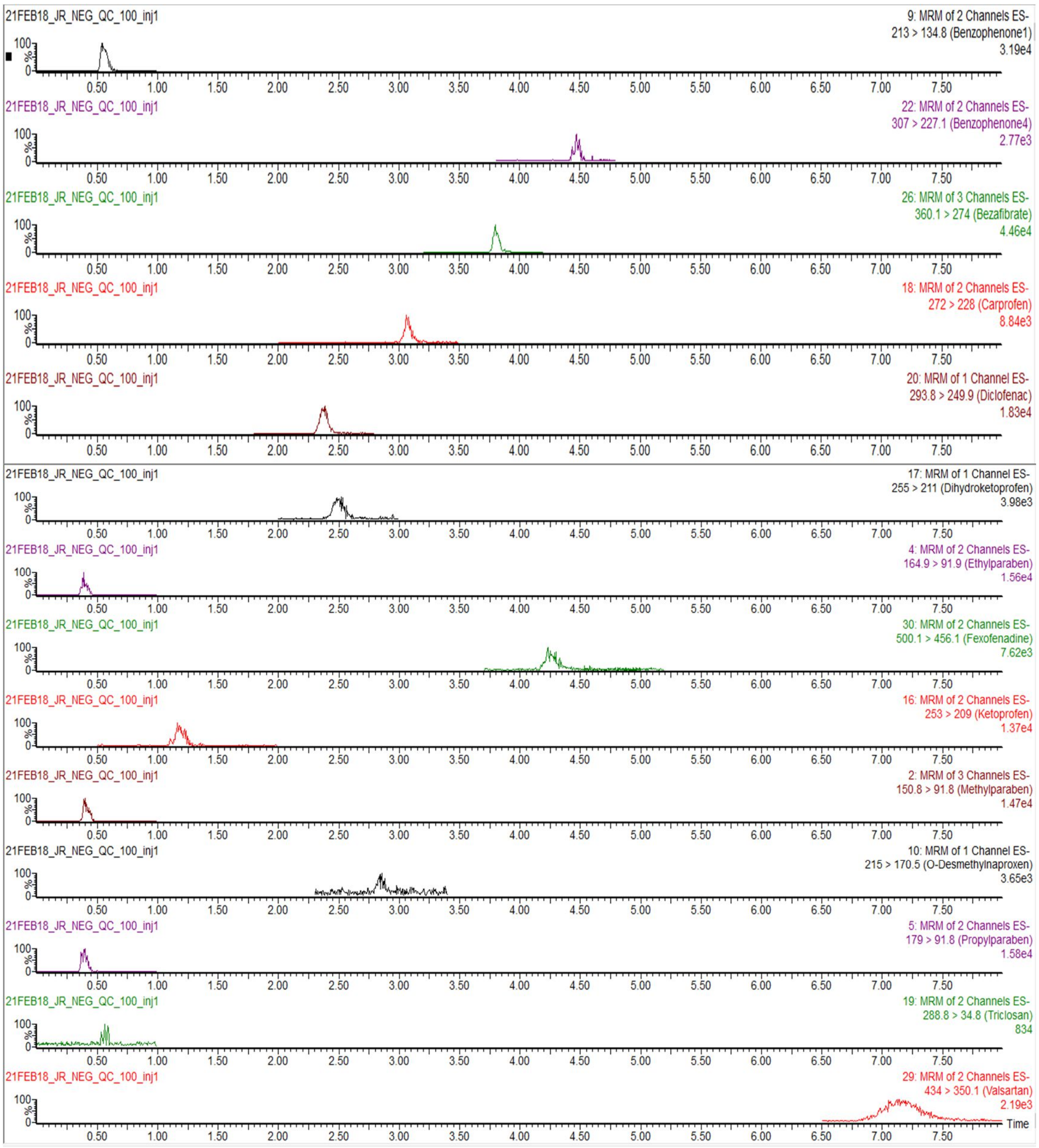

Fig. S4 Mass chromatograms for analytes in the NEG method using their MRM1 transitions

Table S7 Instrument linearity, range, instruments limits of detection and quantification and average relative retention time (t_{rel}) (n=42; semiquantitative compounds are presented in italics)

Analyte	Average t_{rel}	Linear range (µg L^{-1})	R^2	iLOD (µg L^{-1})	iLOQ (µg L^{-1})	Assigned internal standard
**Aminorex**	1.6±0.02	1.5-600	0.997	0.50	1.50	Tetramisole D5
**Anhydroecgonine methylester**	1.0±0.02	0.1-600	0.999	0.05	0.10	Ecgonine methylester D3
**Benzophenone-1**	1.1±0.03	5-1000	0.998	1.00	5.00	Ibuprofen D3
**Benzophenone-4**	11.1±0.02	15-800	0.994	5.00	15.00	Methylparaben 13C6
**Benzoylecgonine**	1.0±0.003	0.05-600	0.998	0.01	0.05	Benzoylecgonine D8
Benzylpiperizine	*7.5±0.02*	*0.5-800*	*0.999*	*0.10*	*0.50*	*Amphetamine D5*
**Bezafibrate**	1.1±0.02	5-1000	0.997	1.00	5.00	Ibuprofen D3
**Buprenorphine**	1.2±0.01	5-400	0.999	1.00	5.00	E2-Mirtazapine D3
Candesartan Cilexetil	*0.9±0.01*	*5-400*	*0.999*	*1.00*	*5.00*	*Gabapentin D4*
**Carbamazepine**	1.0±0.004	0.5-600	0.999	0.10	0.50	Carbamazepine 13C6
**Carbamazepine 10,11 epoxide**	1.3±0.01	0.5-600	0.998	0.10	0.50	Carbamazepine 13C6
Carprofen	*6.1±0.03*	*25-600*	*0.997*	*10.00*	*25.00*	*Ibuprofen D3*
**Citalopram**	0.9±0.02	1.5-400	0.998	0.50	1.50	Citalopram D6
**Clothiniadin**	1.7±0.01	0.5-400	0.998	0.10	0.50	Diazepam D5
**Cocaethylene**	1.0±0.01	0.15-600	0.999	0.05	0.15	Cocaethylene D3
**Cocaine**	1.0±0.02	0.15-600	0.998	0.05	0.15	Cocaine D3
**Codeine**	1.0±0.01	0.15-400	0.998	0.05	0.15	Codeine D6
**Cotinine**	1.0±0.01	0.15-200	0.999	0.05	0.15	Cotinine D3
**Desmethylcitalopram**	1.0±0.01	0.5-400	0.998	0.10	0.50	Codeine D6
DHMA	*0.5±0.03*	*25-600*	*0.993*	*10.00*	*25.00*	*MDMA D5*
**Diazepam**	1.0±0.01	0.15-200	0.999	0.05	0.15	Diazepam D5
**Diazinon**	0.2±0.01	1.5-600	0.999	0.50	1.50	E1-Oxazepam D5
**Diclofenac**	4.7±0.03	5-600	0.997	1.00	5.00	Ibuprofen D3
**Dihydrocodeine**	1.0±0.01	0.15-200	0.997	0.05	0.15	Codeine D6
**Dihydroketoprofen**	4.9±0.03	25-1000	0.995	10.00	25.00	Ibuprofen D3
**Dihydromorphine**	1.2±0.01	5-400	0.998	1.00	5.00	Morphine D3
Diltiazem	*0.8±0.01*	*0.5-800*	*0.999*	*0.10*	*0.50*	*Oxycodone D6*
Duloxetine	*4.5±0.01*	*0.15-600*	*0.997*	*0.05*	*0.15*	*Metazachlor D6*
**E1-10,11-dihydro-10-hydroxycarbamazepine**	0.8±0.01	0.5-200	0.998	0.10	0.5	Carbamazepine 13C6
**E1-Alprenolol**	0.3±0.01	0.25-200	0.997	0.05	0.25	E1-Atenolol D7
**E1-Atenolol**	1.0±0.003	0.75-600	0.998	0.25	0.75	E1-Atenolol D7
**E1-Bisoprolol**	1.1±0.01	0.25-200	0.999	0.05	0.25	E1-Metoprolol D7
**E1-Metoprolol**	1.0±0.02	0.5-200	0.999	0.25	0.5	E1-Metoprolol D7
**E1-Mirtazapine**	1.0±0.01	0.25-100	0.998	0.05	0.25	E1-Mirtazapine D3
**E1-Oxazepam**	1.0±0.01	0.5-200	0.998	0.01	0.50	E1-Oxazepam D5
**E1-Propanolol**	1.0±0.004	1.5-600	0.998	0.50	1.50	E1-Propanolol D7
E1-Tramadol	*0.7±0.01*	*0.25-200*	*0.997*	*0.05*	*0.25*	*MDMA D5*
**E2-10,11-dihydro-10-hydroxycarbamazepine**	1.1±0.01	1.5-800	0.998	0.50	1.50	Carbamazepine 13C6
**E2-Alprenolol**	0.3±0.01	0.25-500	0.998	0.05	0.25	E2-Attenolol D7
**E2-Atenolol**	1.0±0.002	0.75-600	0.999	0.25	0.75	E2-Atenolol D7
**E2-Bisoprolol**	1.0±0.01	0.75-400	1.000	0.25	0.75	E2-Metoprolol D7
**E2-Metoprolol**	1.0±0.01	2.5-200	0.999	1.00	2.5	E2-Metoprolol D7
**E2-Mirtazapine**	1.0±0.01	0.25-200	0.999	0.05	0.25	E2-Mirtazapine D3
**E2-Oxazepam**	1.0±0.004	0.5-400	0.998	0.01	0.50	E2-Oxazepam D5
**E2-Propanolol**	1.0±0.003	0.75-400	0.997	0.25	0.75	E2-Propanolol D7
**Ethylparaben**	1.0±0.03	5-600	0.998	1.00	5.00	Methylparaben 13C6
Fexofenadine	*8.5±0.03*	*25-1000*	*0.993*	*10.00*	*25.00*	*Ibuprofen D3*
**Griseofulvin**	0.8±0.01	1-400	0.997	0.50	1.00	Praziquantrel D11
**Heroin**	1.0±0.02	0.5-800	0.999	0.10	0.50	Heroin D9
**HMA**	2.7±0.01	1-600	0.997	0.50	1.00	MDMA D5
HMMA	*2.1±0.01*	*0.1-200*	*0.999*	*0.05*	*0.10*	*MDMA D5*
**Hydrocodone**	1.0±0.02	5-400	0.997	1.00	5.00	Hydrocodone D6
**Imatinib**	1.7±0.01	0.1-200	0.999	0.05	0.10	Morphine D3
**Imidacloprid**	3.8±0.01	1-200	0.997	0.50	1.00	Metazachlor D6
**Indoprofen**	1.7±0.004	0.5-600	0.999	0.10	0.50	Carbamazepine 13C6
Iopromide	*1.1±0.01*	*10-400*	*0.994*	*5.00*	*10.00*	*Gabapentin D4*
**Ketamine**	1.0±0.01	0.5-600	0.998	0.10	0.50	Ketamine D4
**Ketoprofen**	2.3±0.03	10-1000	0.997	5.00	10.00	Ibuprofen D3

**MDA**	1.0±0.03	0.5-600	0.999	0.10	0.50	MDA D5
**MDMA**	1.0±0.01	0.5-400	0.998	0.10	0.50	MDMA D5
**MDPV**	0.5±0.01	0.05-200	0.998	0.01	0.05	MDMA D5
Memantine	*0.8±0.01*	*0.5-400*	*0.998*	*0.10*	*0.50*	*Codeine D6*
**Mephedrone**	1.0±0.03	0.05-400	0.998	0.01	0.05	Mephedrone D3
**Metazachlor**	1.0±0.02	1.5-400	0.998	0.50	1.50	Metazachlor D6
**Methadone**	1.0±0.01	0.1-600	0.998	0.05	0.10	Methadone D9
**Methamphetamine**	1.0±0.01	0.05-800	0.999	0.01	0.05	Methamphetamine D5
**Methylparaben**	1.0±0.03	5-600	0.997	1.00	5.00	Methylparaben 13C6
**Morphine**	1.0±0.01	5-400	0.998	1.00	5.00	Morphine D3
**Nordiazepam**	1.0±0.01	0.5-200	0.999	0.10	0.50	Nordiazepam D5
**Norephedrine**	1.4±0.01	0.5-600	0.997	0.10	0.50	Flumequine 13C3
**Normorphine**	8.7±0.01	5-800	1.000	1.00	5.00	Metazachlor D6
**Nortriptyline**	1.0±0.01	5-400	0.998	1.00	5.00	Nortryptyline D3
**O-Desmethylnaproxen**	5.6±0.03	75-1000	0.991	25.00	75.00	Ibuprofen D3
**Omeprazole**	0.9±0.01	0.5-400	1.000	0.10	0.50	Quetiapine D8
**Oxadiazon**	0.2±0.03	1.5-400	0.998	0.50	1.50	E1-Oxazepam D5
**Oxycodone**	1.0±0.02	1.5-400	0.998	0.50	1.50	Oxycodone D6
Oxymorphone	*6.8±0.01*	*1.5-600*	*1.000*	*0.50*	*1.50*	*Metazachlor D6*
**Pholcodine**	0.6±0.01	5-200	0.998	1.00	5.00	Gabapentin D4
**Praziquantrel**	1.0±0.01	0.05-400	0.998	0.01	0.05	Praziquantrel D11
**Propylparaben**	1.0±0.03	5-800	0.999	1.00	5.00	Methylparaben 13C6
**Quetiapine**	1.0±0.02	0.1-400	0.998	0.05	0.10	Quetiapine D8
**Risperidone**	0.5±0.01	0.05-400	0.998	0.01	0.05	Gabapentin D4
**Salbutamol**	3.2±0.01	1-200	0.997	0.05	0.10	Diazepam D5
**Sotalol**	0.7±0.01	0.5-400	0.999	0.10	0.50	Gabapentin D4
**Sulphadiazine**	0.9±0.003	0.1-400	0.999	0.05	0.10	E2-Propanolol D7
**Sulphamethoxazole**	0.7±0.02	0.1-1000	0.998	0.05	0.10	Ofloxacin D3
**Sulphapyridine**	0.9±0.003	0.5-200	0.998	0.10	0.50	Benzoylecgonine D8
**Terbutaline**	1.0±0.02	25-800	0.997	10.00	25.00	Fluoxetine D5
**Terbuthylazine**	0.2±0.01	0.15-400	0.999	0.05	0.15	E1-Oxazepam D5
**Tetramisole**	1.0±0.01	1.5-600	0.999	0.50	1.50	Tetramisole D5
Thiamethoxam	*1.1±0.02*	*5-200*	*0.999*	*1.00*	*5.00*	*Quetiapine D8*
Triclosan	*1.1±0.04*	*15-1000*	*0.991*	*5.00*	*15.00*	*Ibuprofen D3*
**Valsartan**	14.2±0.03	15-1000	0.998	5.00	15.00	Ibuprofen D3
**Vardenafil**	0.9±0.01	0.05-400	0.998	0.01	0.05	Gabapentin D4
**Zolpidem**	0.9±0.01	0.5-600	0.999	0.10	0.50	Hydrocodone D6

Table S8 Average absolute recoveries (%) for all analytes analysed in the method, even if not developed further (n=3)

Analyte	River water			Wastewater		
	10 µg L^{-1}	50 µg L^{-1}	200 µg L^{-1}	10 µg L^{-1}	50 µg L^{-1}	200 µg L^{-1}
1,7 dimethylxantine	130.38	51.33	59.65	7320.58	2303.42	318.38
2-Hydroxyibuprofen	18.88	42.19	27.30	16890.07	1991.20	226.19
8-isoF2B	0.00	0.00	101.45	0.00	258.28	95.79
Acetaminophen	106.09	57.33	87.86	8642.40	1522.31	-118.34
Anhydroecgonine methylester	22.99	41.17	48.74	27.85	42.26	45.70
Aminorex	18.02	29.83	32.12	26.73	36.02	41.64
Amphetamine	30.76	59.35	60.77	22.22	28.40	32.16
Azathioprine	0.00	59.68	61.18	30.63	51.60	55.33
Benzophenone-1	29.48	67.94	59.97	75.64	113.44	85.44
Benzophenone-3	9.99	46.16	56.54	-187.79	33.62	54.26
Benzophenone-4	180.31	101.10	79.60	-201.52	145.17	85.40
Benzoylecgonine	50.58	83.03	82.57	47.53	67.41	52.26
Benzylpiperizine	9.12	11.89	23.56	17.58	23.59	32.26
Bezafibrate	57.66	69.82	66.44	67.27	95.59	77.57
Bicalutamide	110.59	72.76	80.32	68.74	13.20	36.45
Buprenorphine	27.70	39.47	56.19	7.11	34.39	40.00
Caffeine	53.67	63.29	85.61	349.13	-34.88	-33.04
Candesartan Cilexetil	20.02	22.92	24.91	4.29	22.82	20.79
Capecitabine	43.75	58.78	63.93	39.60	52.68	55.40
Carbamazepine	48.48	75.00	75.99	50.15	60.47	53.33
Carbamazepine 10,11 epoxide	51.92	75.83	75.37	44.51	65.34	60.60
Carprofen	23.20	68.68	53.29	32.20	61.04	55.09
Chlorpyrifos	-272.63	17.16	21.34	-127.30	16.89	16.63
Cimetidine	21.22	53.14	53.14	62.67	76.14	54.92
Citalopram	34.36	55.57	73.66	11.26	27.94	31.51
Clothiniadin	46.84	69.70	74.66	41.18	49.96	52.04
Cocaethylene	44.81	64.54	71.64	22.24	34.79	38.40
Cocaine	38.96	64.34	69.73	20.35	33.69	37.12
Codeine	31.17	67.54	90.62	166.17	56.83	48.69
Cotinine	26.76	56.98	78.36	34.53	22.55	26.09
Creatinine	-2.85	0.18	0.25	509.83	137.81	-0.38
Cytarabine	-1.58	-0.10	0.52	-1.35	1.73	0.42
Desmethylcitalopram	26.23	39.21	57.52	24.39	46.05	49.95
Desmethylvenlafaxine	23.23	70.99	81.25	73.41	62.60	49.78
Desvenlafaxine	29.49	71.30	79.91	68.06	63.47	47.64
DHMA	11.98	57.91	52.65	95.89	30.14	30.52
Diazepam	56.15	73.41	82.00	41.31	45.23	42.35
Diazinon	35.90	47.89	42.83	40.18	58.87	53.56
Diclofenac	49.80	77.11	67.47	53.64	90.57	75.64
Dihydrocodeine	52.79	70.88	85.94	47.77	56.35	57.27
Dihydroketoprofen	104.48	89.07	69.86	89.69	62.65	71.85
Dihydromorphine	60.70	69.61	75.13	63.09	53.68	48.70
Diltiazem	35.50	54.61	60.67	7.31	18.30	19.58
Duloxetine	9.58	14.48	22.21	0.83	25.53	27.02
E1-10,11-dihydro-10-hydroxycarbamazepine	53.52	79.18	90.76	34.85	59.09	65.89
E1-Alprenolol	48.53	49.23	70.17	19.40	35.84	38.73
E1-Atenolol	46.60	65.81	69.80	-9.66	59.69	53.21
E1-Bisoprolol	38.92	59.11	77.48	34.95	55.82	57.23
E1-Metoprolol	45.99	64.10	77.30	30.54	47.92	51.58
E1-Mirtazapine	42.94	70.68	85.65	17.04	30.42	35.68
E1-Oxazepam	55.45	68.32	67.12	51.25	54.68	51.09
E1-Propanolol	35.49	51.80	62.29	53.72	67.84	63.91
E1-Tramadol	32.06	73.71	78.75	3.51	21.80	25.21
E2-10,11-dihydro-10-hydroxycarbamazepine	50.83	79.73	80.88	47.71	66.05	62.26
E2-Alprenolol	47.04	49.31	64.76	43.13	51.43	48.38
E2-Atenolol	55.74	67.86	75.00	56.15	74.97	61.10
E2-Bisoprolol	40.08	58.74	68.24	55.38	81.21	71.12
E2-Metoprolol	39.85	58.00	69.15	65.86	75.87	69.59
E2-Mirtazapine	54.58	70.00	88.17	27.54	41.84	44.32
E2-Oxazepam	61.50	69.90	67.28	39.36	45.81	44.21
E2-Propanolol	37.05	51.71	65.77	27.51	51.65	53.63
E2-Tramadol	38.70	73.92	75.55	14.33	48.27	42.98
Ephedrine	6.70	8.08	17.89	-23.58	23.67	29.30
Ethylparaben	59.78	87.00	78.39	0.00	127.87	93.85
Fexofenadine	97.97	74.04	67.01	75.84	72.29	68.28
Flufenacet	32.58	46.04	56.01	24.01	31.12	33.15
Fluoxetine	10.94	17.39	29.63	1.70	11.01	12.81
Furosemide	3.53	1.85	1.12	4.84	13.23	5.93
Gabapentin	15.31	0.96	1.40	56.91	9.93	2.54
Gemfibrozil	448.09	68.28	69.93	-519.74	-33.21	15.32

Gliclazide	30.89	59.29	66.52	42.76	51.47	50.69
Griseofulvin	62.76	75.18	95.44	42.34	38.45	49.48
Heroin	28.87	41.02	44.01	14.62	18.22	19.55
HMA	19.75	37.29	44.48	13.54	35.33	36.28
HMMA	45.31	65.03	70.37	44.54	59.97	57.87
HNE-MA	0.00	98.95	64.79	0.00	66.50	79.25
Hydrocodone	42.33	68.62	90.08	85.42	56.98	59.50
Ibuprofen	0.00	116.96	102.88	6291.11	2364.50	562.20
Imatinib	42.45	50.63	55.69	35.65	55.29	49.51
Imazalil sulphate	22.87	48.64	65.00	2.88	12.00	16.60
Imidacloprid	50.06	79.46	87.27	61.58	70.02	66.97
Indoprofen	50.88	68.24	68.29	32.94	44.57	43.67
Iopromide	43.82	71.14	77.87	107.35	87.92	60.68
Irbesartan	47.04	66.20	65.94	44.94	57.07	51.62
Ketamine	49.23	73.70	74.37	28.93	35.63	34.75
Ketoprofen	30.29	87.24	79.39	0.00	83.54	82.07
MDA	22.63	47.14	49.70	29.07	44.36	57.03
MDMA	37.05	64.01	71.57	29.25	38.11	40.49
MDPV	40.48	54.45	68.77	25.76	37.23	37.18
Memantine	38.50	66.94	56.87	43.38	60.00	71.88
Mephedrone	16.15	33.76	41.91	8.52	15.42	21.19
Metazachlor	61.03	74.27	77.81	15.22	39.11	45.58
Methadone	52.35	59.04	68.91	13.90	34.98	36.81
Methamphetamine	20.61	52.81	40.76	14.68	19.52	24.49
Methylparaben	0.00	92.80	76.52	201.09	158.44	97.57
Morphine	49.04	73.39	81.15	150.38	94.67	68.89
N-desmethyl tramadol	19.62	31.91	45.39	37.13	53.31	48.49
N-Guanylurea	-20.70	-3.03	0.11	-6.66	0.04	1.00
Nicotine	-3.60	35.06	40.05	113.06	42.10	28.70
Norcodeine	22.85	21.93	57.17	47.32	30.54	41.45
Nordiazepam	57.15	73.94	81.20	47.52	59.81	56.70
Norephedrine	13.18	16.22	27.49	25.63	30.38	29.36
Norfluoxetine	4.15	7.81	11.92	4.02	13.61	18.90
Normorphine	2.57	31.17	52.07	36.88	51.19	43.43
Noroxycodone	9.93	6.56	17.04	14.64	23.32	26.07
Nortriptyline	21.02	26.26	33.56	13.42	36.69	38.99
O-6-MAM	48.89	83.27	117.30	30.24	55.98	64.73
O-desmethyl tramadol	42.29	73.16	79.00	28.73	48.39	45.60
O-Desmethylnaproxen	0.00	62.40	66.56	0.00	95.71	96.27
Omeprazole	72.68	86.54	75.22	80.08	73.43	54.70
Orlistat	5.60	7.72	11.04	1.73	1.41	1.46
Orlistat	5.18	7.29	10.79	-1.65	1.07	1.41
Oxadiazon	40.26	37.82	39.99	17.69	26.20	25.26
Oxycodone	64.42	74.69	94.18	43.24	41.02	50.38
Oxymorphone	0.00	6.28	17.37	15.14	23.61	28.13
Pholcodine	56.25	76.65	83.27	91.65	72.15	70.54
PMA	30.95	36.10	49.82	0.00	106.43	69.58
Praziquantrel	50.65	78.00	97.41	39.75	51.81	60.21
Pregabalin	0.00	0.00	0.00	7.79	2.51	0.96
Propylparaben	102.92	102.23	86.18	370.29	157.29	106.81
Quetiapine	53.54	67.78	76.65	47.64	55.11	51.89
Ranitidine	28.09	56.33	66.60	240.41	111.69	53.71
Risperidone	50.32	60.62	68.23	43.74	62.09	56.19
Salbutamol	49.45	70.32	76.68	15.78	35.59	37.86
Sertraline	16.33	21.90	31.69	9.63	17.69	17.43
Sitagliptin	-3.95	15.47	24.62	34.13	36.23	32.69
Sotalol	40.86	53.02	68.76	51.81	59.03	52.03
Sulfasalazine	-52.84	12.12	19.67	-13.91	60.34	55.25
Sulphadiazine	35.36	40.65	35.99	40.49	42.05	36.80
Sulphamethoxazole	40.36	59.42	66.30	31.32	51.83	49.21
Sulphapyridine	44.29	62.24	69.41	119.96	78.46	50.55
Terbutaline	12.92	13.09	6.78	10.59	9.46	3.46
Terbuthylazine	40.37	54.88	59.43	30.56	37.61	37.34
Tetramisole	48.29	64.11	74.83	28.05	33.49	33.62
Thiamethoxam	68.36	84.84	102.92	42.42	67.96	74.71
Triclosan	4.71	225.85	45.81	222.34	211.87	46.72
Valsartan	17.95	82.36	72.78	45.83	85.68	72.56
Vardenafil	52.77	67.45	69.07	44.94	71.44	70.32
Venlafaxine	49.69	69.54	73.31	11.59	29.03	29.10
Zolpidem	55.60	76.28	81.95	36.63	63.45	54.91

Table S9 Instrument accuracy and precision assessed over a period of one week (semiquantitative compounds are presented in italics))

Analyte	Accuracy (%)		Intraday precision (% RSD)		Interday precision (Average % RSD)	
	Average (n = 9)	SD	Average (n = 9)	SD	Average (n = 3)	SD
**Aminorex**	99	7.4	4	0.7	3	1.5
**Anhydroecgonine methylester**	101	3.8	2	1.3	2	0.9
**Benzophenone-1**	107	6.9	6	3.8	19	3.4
**Benzophenone-4**	106	26.0	7	1.1	14	7.7
**Benzoylecgonine**	102	5.2	1	1.0	1	0.6
Benzylpiperizine	*99*	*7.2*	*2*	*0.1*	*2*	*0.2*
**Bezafibrate**	123	11.6	7	3.0	16	0.2
**Buprenorphine**	100	8.8	5	1.3	6	1.5
Candesartan Cilexetil	*99*	*9.7*	*5*	*4.3*	*5*	*4.2*
**Carbamazepine**	101	5.9	2	1.0	2	0.6
**Carbamazepine 10,11 epoxide**	97	6.7	3	1.6	3	2.2
Carprofen	*98*	*0.3*	*6*	*2.1*	*15*	*1.6*
**Citalopram**	98	18.0	3	0.4	2	0.4
**Clothiniadin**	97	10.9	2	0.6	2	0.9
**Cocaethylene**	105	7.3	1	0.3	2	0.7
**Cocaine**	103	6.3	3	1.7	3	1.0
**Codeine**	99	13.6	4	0.1	5	1.2
**Cotinine**	99	6.9	3	1.2	2	0.2
**Desmethylcitalopram**	99	13.0	3	1.6	3	0.4
DHMA	*107*	*4.9*	*12*	*9.1*	*7*	*2.8*
**Diazepam**	98	3.2	2	1.2	2	0.7
**Diazinon**	103	4.8	5	3.0	5	2.5
**Diclofenac**	116	0.7	9	6.7	17	0.2
**Dihydrocodeine**	103	9.3	4	1.1	4	0.6
**Dihydroketoprofen**	112	18.3	5	0.8	15	0.4
**Dihydromorphine**	100	13.5	3	1.5	3	2.3
Diltiazem	*106*	*15.8*	*4*	*0.9*	*3*	*0.6*
Duloxetine	*106*	*15.7*	*4*	*1.6*	*4*	*1.6*
**E1-10,11-dihydro-10-hydroxycarbamazepine**	92	5.7	4	0.8	4	3.1
**E1-Alprenolol**	105	8.4	2	1.0	3	0.7
**E1-Atenolol**	96	6.4	1	0.8	2	0.8
**E1-Bisoprolol**	95	4.1	2	1.0	3	1.0
**E1-Metoprolol**	95	3.0	2	0.8	4	1.2
**E1-Mirtazapine**	93	3.4	2	0.0	2	0.3
**E1-Oxazepam**	100	9.1	3	0.8	4	0.3
**E1-Propanolol**	95	9.6	2	1.4	3	0.1
E1-Tramadol	*90*	*6.3*	*3*	*0.1*	*3*	*0.3*
**E2-10,11-dihydro-10-hydroxycarbamazepine**	95	5.0	3	0.5	2	0.4
**E2-Alprenolol**	109	5.9	2	1.2	2	0.8
**E2-Atenolol**	95	4.7	2	1.1	2	0.9
**E2-Bisoprolol**	94	5.7	2	0.6	2	0.5
**E2-Metoprolol**	99	12.4	2	0.1	3	1.8
**E2-Mirtazapine**	97	3.1	2	0.6	2	0.1
**E2-Oxazepam**	101	13.4	2	1.2	2	0.7
**E2-Propanolol**	91	3.5	3	0.6	3	0.1
**Ethylparaben**	103	8.6	8	0.7	11	3.9
Fexofenadine	*104*	*2.8*	*2*	*0.7*	*14*	*12.4*
**Griseofulvin**	97	7.1	4	0.7	4	0.8
**Heroin**	98	14.5	3	0.8	4	0.6

**HMA**	96	12.6	3	1.9	3	1.0
HMMA	*108*	*12.0*	*3*	*2.0*	*2*	*0.9*
**Hydrocodone**	93	18.1	5	1.9	3	0.8
**Imatinib**	105	5.8	3	0.6	3	0.5
**Imidacloprid**	96	10.4	2	1.5	2	0.8
**Indoprofen**	86	6.4	5	4.2	4	3.0
Iopromide	*98*	*17.2*	*4*	*2.4*	*5*	*1.4*
**Ketamine**	103	7.9	1	1.0	3	0.1
**Ketoprofen**	127	9.7	7	5.9	17	3.4
**MDA**	99	10.4	1	0.1	1	0.1
**MDMA**	101	8.5	1	0.6	1	0.4
**MDPV**	104	9.4	3	0.8	3	0.4
Memantine	*100*	*7.6*	*5*	*1.5*	*4*	*1.8*
**Mephedrone**	105	4.1	2	0.5	3	0.2
**Metazachlor**	95	11.6	5	1.9	4	2.0
**Methadone**	104	7.7	3	0.6	2	0.6
**Methamphetamine**	94	10.6	2	0.2	3	0.4
**Methylparaben**	99	8.3	6	0.7	9	5.4
**Morphine**	103	11.0	5	1.6	4	0.3
**Nordiazepam**	102	5.9	2	0.6	2	0.2
**Norephedrine**	102	17.8	7	6.4	3	2.4
**Normorphine**	93	16.6	5	3.4	5	1.8
**Nortriptyline**	97	12.5	3	1.8	4	0.5
**O-Desmethylnaproxen**	142	58.3	12	8.1	11	4.6
**Omeprazole**	106	8.9	1	1.4	2	0.4
**Oxadiazon**	119	11.6	6	2.2	6	2.3
**Oxycodone**	99	12.6	3	1.5	3	1.3
Oxymorphone	*96*	*8.8*	*3*	*2.2*	*3*	*1.1*
**Pholcodine**	100	9.3	4	3.9	4	2.1
**Praziquantrel**	101	5.7	3	0.4	3	0.5
**Propylparaben**	108	12.4	6	1.8	10	1.6
**Quetiapine**	101	15.9	1	0.3	2	0.7
**Risperidone**	102	15.2	2	0.7	2	0.5
**Salbutamol**	103	9.7	1	0.2	1	0.2
**Sotalol**	100	15.4	2	0.3	2	0.4
**Sulphadiazine**	96	4.6	5	1.3	4	0.2
**Sulphamethoxazole**	105	5.1	5	2.4	4	0.1
**Sulphapyridine**	98	5.2	3	1.2	2	1.0
**Terbutaline**	91	55.1	35	38.8	8	7.3
**Terbuthylazine**	106	10.7	5	3.7	4	0.9
**Tetramisole**	96	5.3	4	1.4	4	1.0
Thiamethoxam	*97*	*1.3*	*2*	*1.4*	*4*	*2.0*
Triclosan	*231*	*113*	*10*	*6.5*	*24*	*8.2*
**Valsartan**	105	7.8	8	2.3	15	0.7
**Vardenafil**	96	10.4	2	0.2	2	0.7
**Zolpidem**	104	6.3	8	5.8	5	3.5

Table S10 Average relative recoveries for all analytes analysed in the method, even if not developed further (n=3)

Analyte	River water			Wastewater		
	10 µg L^{-1}	50 µg L^{-1}	200 µg L^{-1}	10 µg L^{-1}	50 µg L^{-1}	200 µg L^{-1}
1,7 dimethylxantine	0.00	110.56	88.46	61862.73	7155.72	1111.20
2-Hydroxyibuprofen	10.39	53.75	32.63	4039.25	1099.68	159.46
8-isoF2B	0.00	0.00	145.67	0.00	234.65	111.65
Acetaminophen	0.00	39.34	81.64	-135882.32	-30190.06	-7853.75
Anhydroecgonine methylester	0.00	47.85	68.24	0.00	110.92	129.70
Aminorex	0.00	61.76	67.19	0.00	30.59	61.38
Amphetamine	0.00	90.65	102.36	0.00	108.83	106.64
Azathioprine	106.73	101.85	143.43	-1.19	-150.11	690.10
Benzophenone-1	0.00	51.02	69.57	128.18	90.18	87.55
Benzophenone-3	-31.17	-742.53	117.79	147.69	-543.44	218.70
Benzophenone-4	0.00	93.04	100.29	0.00	86.48	91.08
Benzoylecgonine	86.46	102.96	112.09	65.07	95.11	104.86
Benzylpiperizine	36.26	26.75	48.54	50.68	46.16	51.13
Bezafibrate	0.00	53.94	78.97	0.00	61.67	75.90
Bicalutamide	136.54	56.24	94.07	173.08	12.35	57.55
Buprenorphine	39.34	53.10	65.38	68.01	56.16	57.79
Caffeine	103.80	94.05	105.17	-4020.34	-1532.72	-125.69
Candesartan Cilexetil	72.91	40.60	27.88	89.90	72.68	49.45
Capecitabine	35.50	23.22	19.76	8.89	61.75	80.55
Carbamazepine	76.65	93.82	99.92	80.14	89.22	96.79
Carbamazepine 10,11 epoxide	68.68	92.43	98.12	64.14	99.44	107.35
Carprofen	0.00	63.14	61.33	49.19	36.74	56.48
Chlorpyrifos	0.00	22.56	16.56	-220.00	42.68	38.43
Cimetidine	102.77	122.56	57.50	2.43	-190.70	290.87
Citalopram	94.56	76.60	78.02	0.00	45.23	68.85
Clothiniadin	59.56	89.64	86.44	92.75	117.81	101.84
Cocaethylene	0.00	83.07	96.29	93.51	93.36	96.83
Cocaine	0.00	88.43	89.94	0.00	86.68	92.82
Codeine	160.55	96.79	97.11	0.00	52.91	84.02
Cotinine	123.95	102.48	99.31	0.00	65.66	89.11
Creatinine	0.82	0.41	0.09	-42.97	140.16	-3.23
Cytarabine	-333.81	-57.39	-10.08	-1336.61	-226.00	-43.16
Desmethylcitalopram	94.75	76.71	58.03	138.63	133.54	103.76
Desmethylvenlafaxine	111.68	109.38	77.72	-351.42	-139.77	65.46
Desvenlafaxine	124.62	111.71	80.87	-744.51	-203.87	69.80
DHMA	0.00	85.75	77.30	0.00	46.58	61.28
Diazepam	71.10	87.78	92.16	91.03	96.84	90.29
Diazinon	77.71	67.32	49.94	120.15	141.31	104.41
Diclofenac	0.00	84.73	79.95	87.30	54.06	82.71
Dihydrocodeine	87.90	82.89	88.82	0.00	77.22	83.97
Dihydroketoprofen	0.00	74.47	97.87	0.00	38.87	75.01
Dihydromorphine	91.75	93.84	85.74	111.26	85.26	68.65
Diltiazem	84.26	72.69	69.02	109.47	58.05	36.55
Duloxetine	27.17	19.07	26.29	55.86	46.38	44.05
E1-10,11-dihydro-10-hydroxycarbamazepine	0.00	96.78	125.36	0.00	98.52	125.92
E1-Alprenolol	0.00	69.32	96.88	0.00	60.72	70.67
E1-Atenolol	82.72	98.37	98.16	0.00	93.08	95.97
E1-Bisoprolol	77.17	83.75	101.15	65.40	92.01	109.38
E1-Metoprolol	72.74	88.67	100.40	61.93	81.10	97.81
E1-Mirtazapine	70.48	80.13	90.37	0.00	75.60	81.29
E1-Oxazepam	75.63	91.42	89.28	63.80	95.14	78.04
E1-Propanolol	79.59	89.97	96.46	72.60	86.76	96.99
E1-Tramadol	60.43	103.27	101.81	0.00	26.29	48.46
E2-10,11-dihydro-10-hydroxycarbamazepine	94.08	101.80	107.65	122.08	104.43	113.97
E2-Alprenolol	58.45	63.54	85.62	0.00	70.21	73.95
E2-Atenolol	75.10	89.05	98.99	0.00	96.51	94.27
E2-Bisoprolol	76.18	83.58	99.03	0.00	86.88	93.79
E2-Metoprolol	64.96	79.82	100.46	100.44	84.47	93.59
E2-Mirtazapine	66.29	83.89	90.74	0.00	80.17	80.51
E2-Oxazepam	95.12	100.06	87.86	69.27	91.51	75.21
E2-Propanolol	96.41	91.41	91.35	0.00	86.05	88.21
E2-Tramadol	80.74	107.43	110.59	0.00	31.50	54.53
Ephedrine	37.40	40.14	48.09	-100.62	36.96	60.39
Ethylparaben	78.00	89.67	106.35	0.00	82.98	91.94
Fexofenadine	0.00	97.51	80.65	0.00	34.79	64.93
Flufenacet	325.72	54.85	66.33	179.85	73.90	66.89
Fluoxetine	58.20	51.17	51.08	-54.15	29.26	40.06
Furosemide	132.16	116.42	0.00	133.15	114.80	0.00
Gabapentin	92.70	20.31	6.49	-14983.04	-3829.28	-851.19
Gemfibrozil	525.36	-467.06	53.79	1561.83	-1579.26	-116.71

Gliclazide	-2.29	-51.17	-2459.15	-3.65	-57.30	-2526.93
Griseofulvin	88.37	82.35	94.53	96.58	69.61	84.48
Heroin	84.28	86.55	93.54	0.00	89.56	84.95
HMA	66.00	53.80	55.72	80.22	78.37	71.47
HMMA	69.25	83.71	90.31	159.22	150.74	130.34
HNE-MA	0.00	37.60	100.26	0.00	79.46	150.77
Hydrocodone	103.31	88.79	89.88	0.00	65.79	81.97
Ibuprofen	0.00	138.19	149.28	0.00	699.53	130.70
Imatinib	76.30	63.24	64.91	125.66	96.07	79.54
Imazalil sulphate	0.00	70.40	93.45	0.00	30.49	47.19
Imidacloprid	103.26	108.38	101.51	120.74	160.62	129.84
Indoprofen	63.68	84.53	88.63	66.73	66.47	76.99
Iopromide	95.99	95.23	74.24	0.00	175.85	149.24
Irbesartan	0.00	47.86	72.17	21.78	42.36	49.64
Ketamine	68.73	95.64	110.65	93.57	109.17	108.44
Ketoprofen	0.00	64.57	122.99	0.00	48.64	86.97
MDA	62.42	83.74	64.83	108.12	82.64	75.40
MDMA	67.35	83.44	91.82	66.68	89.17	91.51
MDPV	66.38	70.30	88.26	83.99	92.22	83.60
Memantine	69.26	115.51	101.28	0.00	151.50	170.19
Mephedrone	0.00	61.23	90.98	0.00	53.90	70.42
Metazachlor	99.19	99.20	89.86	63.17	95.55	89.13
Methadone	0.00	69.36	88.92	0.00	73.43	83.11
Methamphetamine	0.00	79.09	79.98	96.86	84.54	87.58
Methylparaben	0.00	98.28	100.15	0.00	103.82	100.58
Morphine	99.32	100.70	93.59	0.00	108.61	94.87
N-desmethyl tramadol	46.87	64.34	97.27	95.26	263.88	215.37
N-Guanylurea	-95790.65	-1016.78	-230.66	-34802.26	-366.18	-81.29
Nicotine	-2.74	-21555.17	75.52	-178.01	-56282.76	146.11
Norcodeine	21.20	24.01	58.83	-31.70	43.53	60.32
Nordiazepam	75.55	83.52	93.57	101.73	94.51	92.24
Norephedrine	88.42	83.33	73.82	84.76	69.04	62.40
Norfluoxetine	7.81	21.26	20.54	-106.22	39.69	60.90
Normorphine	45.79	46.77	60.91	0.00	71.90	60.60
Noroxycodone	-12.32	5.09	16.85	-13.43	49.76	47.73
Nortriptyline	63.23	62.55	59.25	88.77	65.00	57.94
O-6-MAM	0.00	193.32	272.59	-686.70	379.84	320.14
O-desmethyl tramadol	110.24	148.92	170.38	2.61	235.49	203.46
O-Desmethylnaproxen	0.00	60.71	82.75	0.00	54.04	121.92
Omeprazole	100.52	105.24	78.25	0.00	145.58	119.15
Orlistat	0.00	0.57	12.48	-197.37	-7.50	0.00
Orlistat	17.62	13.13	13.56	0.00	7.65	5.33
Oxadiazon	87.36	60.95	46.89	96.81	75.82	50.67
Oxycodone	84.82	93.36	93.98	123.41	95.08	93.49
Oxymorphone	13.75	8.97	17.67	55.07	41.19	40.08
Pholcodine	93.94	89.51	85.71	121.07	103.92	100.87
PMA	270.74	191.36	139.39	0.00	225.09	150.46
Praziquantrel	72.68	85.39	96.94	90.38	93.18	103.15
Pregabalin	0.00	0.00	0.00	-11.51	-4.66	0.58
Propylparaben	24.53	86.55	108.29	56.37	100.95	106.58
Quetiapine	77.32	79.95	88.81	138.45	95.59	83.75
Ranitidine	0.00	117.09	118.99	-249112.12	-38521.13	-7552.42
Risperidone	87.66	76.86	77.12	96.91	97.51	85.67
Salbutamol	99.98	95.80	77.40	0.00	85.99	102.89
Sertraline	14.52	36.32	46.60	-218.14	-15.59	22.58
Sitagliptin	57.64	28.14	30.13	214.43	87.00	57.79
Sotalol	90.97	74.04	69.74	0.00	149.67	142.30
Sulfasalazine	0.00	0.00	20.86	0.00	39.54	49.62
Sulphadiazine	53.63	66.77	49.83	73.20	74.72	62.25
Sulphamethoxazole	114.69	104.15	138.23	0.00	120.45	116.93
Sulphapyridine	87.66	78.41	95.06	0.00	121.17	101.46
Terbutaline	98.55	98.93	0.00	82.53	85.55	0.00
Terbuthylazine	81.22	75.56	68.91	94.24	92.72	73.26
Tetramisole	0.00	89.42	103.01	0.00	93.17	92.79
Thiamethoxam	105.91	112.66	121.21	130.31	168.11	149.41
Triclosan	0.00	129.08	51.90	0.00	40.80	30.57
Valsartan	0.00	75.95	84.27	69.78	75.52	78.20
Vardenafil	88.49	83.54	78.09	97.10	107.83	105.12
Venlafaxine	124.94	97.53	75.43	-1022.94	-43.87	67.67
Zolpidem	77.51	91.88	91.53	85.35	135.28	116.15
